# Tests for associations between sexual dimorphism and patterns of quantitative genetic variation in the water strider, *Aquarius remigis*

**DOI:** 10.1038/s41437-023-00626-5

**Published:** 2023-05-29

**Authors:** Daphne J. Fairbairn, Derek A. Roff, Matthew E. Wolak

**Affiliations:** 1 Department of Evolution, Ecology and Organismal Biology, University of California, 2710 Life Science Bldg., Riverside, CA 92521 USA; 2 Department of Biological Sciences, Auburn University, 306 Funchess Hall, Auburn, AL 36849 USA; 3 Present Address: Department of Evolution, Ecology and Organismal Biology, University of California, 2710 Life Science Bldg., Riverside, CA 92521 USA

**Keywords:** Evolutionary genetics, Heritable quantitative trait

## Abstract

The evolution of sexual dimorphisms requires divergence between sexes in the evolutionary trajectories of the traits involved. Discerning how genetic architecture could facilitate such divergence has proven challenging because of the difficulty in estimating non-additive and sex-linked genetic variances using traditional quantitative genetic designs. Here we use a three-generation, double-first-cousin pedigree design to estimate additive, sex-linked and dominance (co)variances for 12 traits in the water strider, *Aquarius remigis*. Comparisons among these traits, which have size ratios ranging from 1 to 5 (larger/smaller), allow us to ask if sexual dimorphisms are associated with characteristic patterns of quantitative genetic variation. We frame our analysis around three main questions, derived from existing theory and empirical evidence: Are sexual dimorphisms associated with (1) lower additive inter-sex genetic correlations, (2) higher proportions of sex-linked variance, or (3) differences between sexes in autosomal additive and dominance genetic variances? For questions (1) and (2), we find weak and non-significant trends in the expected directions, which preclude definitive conclusions. However, in answer to question (3), we find strong evidence for a positive relationship between sexual dimorphism and differences between sexes in proportions of autosomal dominance variance. We also find strong interactions among the three genetic components indicating that their relative influence differs among traits and between sexes. These results highlight the need to include all three components of genetic (co)variance in both theoretical evolutionary models and empirical estimations of the genetic architecture of dimorphic traits.

## Introduction

The quantitative genetic analysis of non-additive and sex-linked effects is challenging and consequently has been tackled in relatively few cases. However, as described below, theoretical and empirical studies suggest that both types of effects may play important roles in the evolution and maintenance of sexual dimorphisms. In 2006, Fairbairn and Roff proposed a three-generation pedigree breeding design and method of analysis that enables researchers to estimate the genetic (co)variance components due to both autosomal and sex-linked effects (Fairbairn and Roff 2006; Meyer 2008; Kaufmann et al. 2021). We apply this method to ask if sexually dimorphic traits have characteristic patterns of autosomal and sex-linked (co)variances when compared to less dimorphic traits within the same species.

We frame our analyses around three main questions suggested by available theory and empirical data:

Question 1: Are sexual dimorphisms associated with lower additive inter-sex genetic correlations?

Question 2: Are sexual dimorphisms associated with higher proportions of sex-linked variance?

Question 3: Are sexual dimorphisms associated with differences between sexes in autosomal additive and dominance genetic variances?

These three questions arise because, from the perspective of quantitative genetics, divergent evolution of the sexes cannot occur if the genetic basis of the trait is identical in the two sexes. Specifically, trait values can follow divergent evolutionary trajectories in males and females only if the overall additive inter-sex genetic correlation, *r*_*A*_ (often referred to as *r*_*MF*_ or _*FM*_) is less than 1.0 for the diverging traits, or if the amount of additive genetic variance for the trait differs between sexes (Lande 1980; Lynch and Walsh 1998; Wyman et al. 2013). A corollary of the first mechanism is that disruptive selection favoring different values in the two sexes would be expected to favor the reduction of *r*_*A*_. This has led to the prediction that *r*_*A*_ should be lower for sexually dimorphic traits than for traits with the same optima in both sexes (Lande 1980; Bonduriansky and Rowe 2005; Fairbairn and Roff 2006). In accordance with this prediction, comparisons among traits within populations (Bonduriansky and Rowe 2005; Fairbairn 2007; Cox et al. 2017), as well as a meta-analysis across trait types and species (Poissant et al. 2010), have revealed significant negative correlations between *r*_*A*_ and the magnitude of sexual dimorphism. However, it is important to note that a similar pattern could evolve if traits with initially low *r*_*A*_ simply respond faster or further in response to selection favoring different optimal trait values in the two sexes. In support of this, simulations by Reeve and Fairbairn (2001) found little change in *r*_*A*_ over the evolutionary trajectory from monomorphism to dimorphism. A negative association between *r*_*A*_ and the magnitude of sexual dimorphism on its own does not distinguish between these two causative mechanisms.

Question 2 addresses one mechanism by which a reduction in *r*_*A*_ could be achieved. In species with chromosomal sex determination, one mechanism would be the transfer of genes with sexually antagonistic fitness effects from autosomes to sex chromosomes (Rice 1984; Chenoweth et al. 2008; Boulton et al. 2016; Rowe et al. 2018; Kaufman et al. 2021), and particularly to the X or Z chromosome (Charlesworth and Charlesworth 1980; Rice 1984; Patten and Haig 2009; Mank 2009). Because the additive genetic variance associated with sex-linked genes differs between sexes (Lynch and Walsh 1998; Fairbairn and Roff 2006), this mechanism would be expected to reduce *r*_*A*_.

Question 3 is suggested, in part, by the hypothesis that sexual dimorphism could arise if one sex has more additive genetic variance for the trait than the other, and thus responds more rapidly to selection (Lynch and Walsh 1998). Sex-linked additive effects would contribute to such a difference, but autosomal genes are also expected to have sex-specific effects (Rhen 2000; Fairbairn and Roff 2006; Connallon and Clark 2010; Fry 2010; Postma et al. 2011). Sex-specific patterns of autosomal gene expression (e.g., Weiss et al. 2006; Ellegren and Parsch 2007; Mank 2009; Carreira et al. 2009; Wyman et al. 2013; Gilks et al. 2014; Cheng and Kirkpatrick 2016; Han and Dingemanse 2017; Wright et al. 2018) provide a likely mechanism for this, but to what extent such patterns affect patterns of genetic variance is not known. A recent study of the phenotypic effects from knockout mutations in mice has shown that sex-specific networks of gene expression are common across many different types of traits and are not restricted to traits that have sexually dimorphic phenotypes (Van der Bijl and Mank 2021). These results suggest that cryptic sexual differences in gene expression (i.e., differences that do not lead to phenotypic differences) could provide a previously unappreciated reservoir for the rapid divergence of genetic variances between the sexes and response to sexually antagonistic selection (*op.cit*.).

Numerous studies have shown differences in additive genetic variances between the sexes (e.g., Jensen et al. 2003; Wyman and Rowe 2014; Gilks et al. 2014; Ge et al. 2017; Han and Dingemanse 2017; Kralj-Fiser et al. 2019), but few of these have distinguished autosomal from sex-linked variances. Our review of the literature discovered only seven such studies (Supplementary Table S1 and Supplementary Fig. S1). One study of seed beetles, *Callosobruchus maculatus*, provided 95% confidence limits for the estimates, and in this study, the proportion of autosomal variance was much higher in females (confidence intervals did not overlap), whereas the proportions of sex-linked variance had broadly overlapping confidence intervals (Kaufmann et al. 2021). Three other studies included standard errors for their estimates, but in all cases, approximate confidence intervals of ±2SE overlapped, often broadly. Taken together, the seven studies revealed no overall pattern of differences between sexes (Supplementary Fig. S1), with ranges across traits typically broadly overlapping for both variance components. Thus, the question of whether the proportion of autosomal additive genetic variance tends to differ between sexes remains open, as does the question of whether such differences are associated with sexual dimorphisms.

In addition to additive genetic variances, non-additive allelic interactions, particularly sex-specific patterns of allelic dominance, may also be important in the evolution and maintenance of sexual dimorphisms (Kidwell et al. 1977; Rice 1984; Fry 2010; Arnqvist et al. 2014; Spencer and Priest 2016; Grieshop and Arnqvist 2018; Connallon and Chenoweth; 2019; Kaufman et al. 2021). Non-additive allelic interactions contribute to both additive and dominance genetic variances (Cheverud and Routman 1995; Hill et al. 2008), and hence sex-specific patterns of allelic dominance may contribute to sex differences in both variance components. Significant dominance effects and dominance variances have been documented for many traits and species (Crnokrak and Roff 1995; Roff and Emerson 2006; Wolak and Keller 2014), but few studies have related these to sexual dimorphism. Grieshop and Arnqvist (2018) found strong dominance allelic effects, including strong patterns of sex-specific dominance, for fitness in the seed beetle, *Callosobruchus maculatus*. In the same species, Kaufmann et al. (2021) found sex-specific dominance variance for body size and demonstrated, through a selection experiment, that this facilitated response to selection for sexual dimorphism. Wolak (2013), using line cross-analysis, found a positive correlation between sexual dimorphism and the magnitude of autosomal dominance effects for a suite of size traits in *Aquarius remigis*. We ask if a similar pattern holds for autosomal dominance variances.

In the following sections, we use this framework of three questions to determine if sexual dimorphisms are associated with characteristic patterns of quantitative genetic (co)variation. We use the water strider, *Aquarius remigis*, as our study animal and apply the breeding design of Fairbairn and Roff (2006) to estimate the autosomal additive, autosomal dominance and sex-linked additive (co)variances for a suite of twelve body size traits that differ greatly in the magnitude of sexual dimorphism. Comparisons among traits are then used to test for the suggested relationships between the genetic parameters and the magnitude of sexual dimorphism.

## Materials and methods

### Study animal

*Aquarius remigis* are surface-dwelling semiaquatic bugs (Hemiptera, Gerridae), commonly called water striders, found on streams and small rivers across much of sub-arctic North America (Preziosi and Fairbairn 1992). Adults are primarily wingless (>99% in most populations), and following the precedent of previous studies of the genetics of body size variation in this species (Preziosi and Roff 1998; Fairbairn 2007), our analysis includes only wingless individuals.

Females average 5–11% longer than males, but the magnitude and direction of sexual dimorphism varies greatly among body components (Table 1 and Supplementary Figs. S2 and S3). This reflects the underlying pattern of sexually antagonistic selection: fecundity selection favors longer abdomens in females whereas sexual selection favors longer genitalia in males (Preziosi et al. 1996; Preziosi and Fairbairn 1996, 1997, 2000; Ferguson and Fairbairn 2000; Sih et al. 2002; Bertin and Fairbairn 2005).

**Table 1:** Trait means, standard deviations, size ratios and sexual dimorphism indices for all adults in the full pedigree.

Trait	Abbreviation	Male		Female		Size ratio	SDI^a^
		Mean	Stdev	Mean	Stdev	F/M	
Head length	L.Head	1.517	0.064	1.545	0.066	1.018	0.018
Thorax length	L.Thorax	6.087	0.256	6.279	0.268	1.031	0.031
Abdomen length	L.Abdomen	3.925	0.161	6.433	0.260	1.639	0.639
Abdomen width	W.Abdomen	2.415	0.151	2.759	0.157	1.142	0.142
Front femur length	L.Frontfemur	4.259	0.186	4.128	0.178	0.969	−0.032
Front femur width	W.Frontfemur	0.636	0.045	0.513	0.040	0.807	−0.240
Middle femur length	L.Midfemur	9.172	0.459	8.810	0.403	0.961	−0.041
Hind femur length	L.Hindfemur	8.661	0.439	8.181	0.377	0.945	−0.059
Length of outer margin of segment 7	L.Seg7marg.	1.776	0.100	1.567	0.071	0.883	−0.133
Distance between tips of connexival spines	SpineDist.	1.599	0.146	1.395	0.110	0.872	−0.146
Length of segment 8	L.Seg8	1.242	0.117	0.550	0.054	0.443	−1.257
Length of segments 9 and 10	L.Seg9.10	1.442	0.090	0.287	0.048	0.199	−4.020

All measurements are in mm.

^a^Sexual dimorphism index, SDI = (size of larger sex/size of smaller sex) – 1, arbitrarily set as negative when males are larger than females.

Lovich and Gibbons (1992). For the statistical analyses, the SDI was calculated based on the 12 subdivisions of the data (see Methods).

Sex is determined chromosomally in *A. remigis*, with females having two X chromosomes, and males one (Fairbairn et al. 2016). The absence of a Y chromosome simplifies the quantitative genetic analyses and interpretation of sex-linked effects because all sex-linkage can be ascribed to X-linked effects. In addition, the potential for X-linked effects is high because the X chromosome is the largest chromosome in the karyotype, averaging 13% larger than the largest of the 10 autosomes and comprising 14% of the haploid chromosome complement (Fairbairn et al. 2016).

Previous studies of the additive genetic (co)variance structure of morphological traits in *A. remigis* established that the lengths of the somatic and genital components have significant heritabilities and inter-sex additive genetic correlations (Preziosi and Roff 1998; Fairbairn 2007). In addition, full-sib estimates of *r*_*A*_ showed the predicted negative correlation with the magnitude of dimorphism for a suite of 5 independent size traits (Fairbairn 2007). The study described herein greatly extends this work by increasing the number of traits included and by providing estimates of autosomal additive, autosomal dominance and sex-linked (co)variances.

### Analysis of the genetic architecture of sexual dimorphism

The majority of experimental designs or analyses of unmanipulated populations cannot estimate both autosomal dominance and additive X-linked variances and the former are typically assumed to be zero and omitted from the analysis (see “Dominance and maternal variances” in Supplementary Information and Supplementary Table S1). Maternal effects are also frequently omitted. This can be justified because maternal effects are most frequently found during juvenile or larval stages and are greatly reduced or absent in adults (see Supplementary Fig. S4 and associated references).

**Fig. 1:**
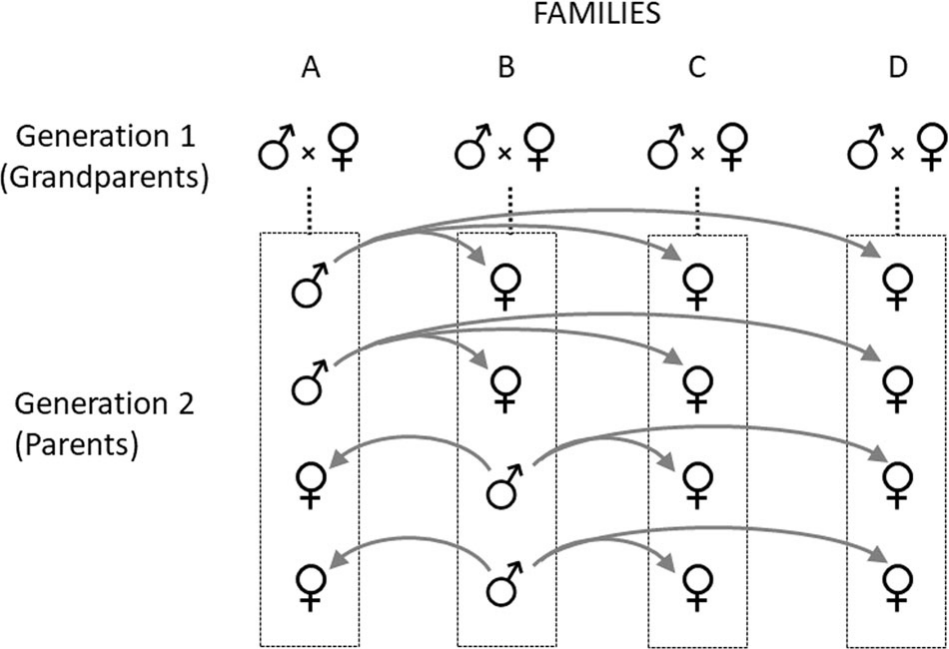
Illustration of a single breeding unit within the pedigree design. Each three-generational breeding unit includes four unrelated grandparental pairs (grandparental generation), plus four of their full-sib offspring (parental generation) with sex ratios as indicated, plus the offspring (F1 generation) of a prescribed set of matings among the families in generation 2. To produce the F1 generation (not shown), four sibs from each family are crossed as indicated by the gray arrows. Brothers from family A mate with sisters from families B, C and D. Similarly, brothers from family B mate with sisters from families A, C and D. This design produces a gradation of relationships in the F1 generation including full sibs, half sibs, first cousins and double-first cousins.

Our analysis uses the animal model (Lynch and Walsh 1998) based on a pedigree from a three-generation, double-first cousin breeding design developed specifically to estimate the autosomal additive and X-linked additive variances (Fig. 1; Fairbairn and Roff 2006). Simulations have shown that, when analyzed using the animal model approach, this design produces more precise and unbiased estimates of sex-specific additive (co)variances than alternate approaches such as mean squares (Meyer 2008). This analysis also estimates the sum of dominance and maternal variances but cannot adequately estimate each separately (Meyer 2008). As noted above, maternal effects are often absent in adults and on average account for a much lower proportion of phenotypic variance than do dominance effects: 3% (Supplementary Fig. S1) versus 14% (Wolak and Keller 2014). Given the potentially small contribution of maternal effects relative to dominance, we ascribe the dominance + maternal genetic variance component to dominance variance.

### Experimental protocols

The experiment was conducted in three consecutive, independent replicates (Table 2). Each replicate was initiated using eggs collected from laboratory-acclimated adults collected from Rattlesnake Creek, in Santa Barbara County, California. Individuals reared from these eggs formed the GP generation. Stratified random sampling and randomized blocked rearing protocols were used to minimize the possibility that environmental effects could bias our estimates, and all rearing was done in environmental chambers set at 25 °C, under a light regime of 14hL:10hD. Adults were preserved in 70% ethanol for later photographing and measurement. Detailed descriptions of the founding samples (Supplementary Table S2), rearing regimes and experimental protocols are given in the Supplementary Information. The final data included individuals contributing to 36 breeding units plus additional full-sib families from the GP and P generations (Table 2).

**Table 2:** Total numbers of measured adults in the pedigree^a^.

Replicate	No. of breeding units	Males				Females				Total
		GP	P	F1	Total	GP	P	F1	Total	
1	15	58	163	218	439	61	204	218	483	922
2	6	34	163	322	519	38	209	309	556	1075
3	15	63	406	195	664	64	408	192	664	1328
Total	36	155	732	735	1622	163	821	719	1703	3325

^a^Includes 36 breeding units plus extra full-sib families contributing to the GP and P generations.

### Traits and measurement protocols

All traits (Table 1) are linear measures taken in ventral view, and none are linear components of any other. Photographs of males and females showing the somatic and genital measurements are shown in Supplementary Figs. S2 and S3 (see also Fairbairn 2005, 2007, 2016; Fairbairn et al. 2003). Somatic measurements include the lengths of the head, thorax, abdomen and femora of the front, middle and hind legs, plus the width of the abdomen and the width of the front femur at its widest point. Two additional somatic measurements capture sex-specific morphologies characterizing the last abdominal segment, segment 7. This segment is elongated in males to facilitate clasping females, and the shape of the distal ventral margin is modified to facilitate movement of the phallus (Fairbairn et al. 2003). In addition, the lateral margins extend backward to form a pair of connexival spines that flank the genital segments and, in some species, aid females in repelling male mating attempts (Arnqvist and Rowe 2002). To characterize size dimorphism in segment 7, we measured the length of the outer (lateral) margin of segment 7 and the distance between the tips of the connexival spines.

As in other gerrids (Rowe and Arnqvist 2012), the genitalia of *A. remigis* have evolved through sex-specific modifications of the three terminal segments of the primitive abdomen (segments 8, 9 and 10) (Fairbairn et al. 2003). To capture sexual differences in genital size for homologous genital components, we measured the length of segment 8 and the combined length of segments 9 and 10.

All measurements were taken under a dissecting microscope, following standard protocols established in previous studies (e.g., Preziosi and Fairbairn 1996, 1997; Bertin and Fairbairn 2007). A detailed description can be found in Supplementary Information.

Our questions pertain to the magnitude of sexual dimorphism, without regard for which sex is larger. To quantify this, we used the following index: (size of the larger sex divided by size of the smaller sex) – 1, which has the conceptual advantage of being zero for monomorphic traits. This is the absolute value of the dimorphism index (SDI) of Lovich and Gibbons (1992), which is arbitrarily assigned to be negative if males are the larger sex. Hence, our index is abs(SDI).

For the analysis of genetic variance structure, we standardized the data for each trait across the entire data set, including both sexes and all generations and replicates, by subtracting the mean and dividing by the standard deviation. This has the effect of removing any possible confounding influence of differences among traits in means and phenotypic variances.

### Estimation of (co)variances

We partitioned phenotypic (co)variances for each sex into autosomal additive and autosomal dominance (co)variances and sex-linked additive variance using quantitative genetic linear mixed models, commonly known as ‘animal models’ (Lynch and Walsh 1998), as proposed by Fairbairn and Roff (2006) and implemented in the asreml-r software package. Matrix inverses for each genetic relatedness matrix, required to estimate genetic (co)variances, were formed using the nadiv (Wolak 2012, 2013) package for R. Models that included sex-linked dominance variance failed to converge, but the models did converge when sex-linked dominance was omitted from the estimation. The experimental procedure is, in principle, capable of estimating this variance component (Meyer 2008), indicating that the failure to converge was most likely due to the sex-linked dominance being too small to estimate. In such cases, the algorithm tends to become unstable and may not be able to converge. We therefore excluded this variance component from our final models.

The inverse of the Average Information matrix was used to obtain approximate standard errors for (co)variances from the model and, in conjunction with the delta method (Lynch and Walsh 1998, Appendix 1), to obtain approximate standard errors on linear functions of the model estimated (co)variances (e.g., variances expressed as proportions of phenotypic variance and correlations).

### Genetic independence of traits

To determine if all of the traits can legitimately be considered as different traits at the genetic level, we did an initial analysis of pairwise genetic correlations among traits within each sex (Supplementary Tables S3 and S4). All were significantly less than 1.0, and almost a third (43 of 132) did not differ significantly from 0. All of the significant correlations were positive but the magnitudes averaged only 0.47 in males and 0.32 in females, and only 5 exceeded 0.8. These correlations show that there is adequate scope for separate evolution of the traits and therefore we retained all 12 traits in our analyses.

### Independence of estimates of abs(SDI)

Because all traits are morphological traits, it is likely that they are phenotypically correlated within individuals, thus possibly making estimates of SDI not statistically independent. To eliminate this possibility, we randomly assigned individuals to one of 12 equal-sized subsets and estimated mean values for only one trait per subset (thus a subset for head width, another for thorax, etc.). Our estimates of abs(SDI) for each trait are derived from these subsets and so the estimates for the 12 traits are statistically independent. The reported results are based on these values. (We also did the analysis using the trait values from the undivided data set and this did not change any of our conclusions.)

### Model validation

We began our estimation of genetic (co)variances with a preliminary analysis of each sex separately, incorporating generation and replicate as fixed effects and the additive, dominance and sex-linked variance components as random effects. Model convergence was obtained for all 12 traits for both sexes. However, the sex-linked additive genetic variance in males, calculated as a proportion of the total phenotypic variance, averaged only 0.34% compared to 9.91% in females. The very low value in males suggested that the sex-linked additive covariance was probably insignificant or very small. To test this, we ran the full model in which males and females were incorporated into a single model but treated separately by considering them as separate environments, as suggested by Falconer (1952):

$$\begin{array}{l}\left[ {\begin{array}{*{20}{c}} {V_{pM}} & {Cov_{pMF}} \\ {Cov_{pMF}} & {V_{pF}} \end{array}} \right] = \left[ {\begin{array}{*{20}{c}} {V_{aM}} & {Cov_{aMF}} \\ {Cov_{aMF}} & {V_{aF}} \end{array}} \right] + \left[ {\begin{array}{*{20}{c}} {V_{dM}} & {Cov_{dMF}} \\ {Cov_{dMF}} & {V_{dF}} \end{array}} \right]\\ + \left[ {\begin{array}{*{20}{c}} {V_{xM}} & {Cov_{xMF}} \\ {Cov_{xMF}} & {V_{xF}} \end{array}} \right] + \left[ {\begin{array}{*{20}{c}} {V_{eM}} & 0 \\ 0 & {V_{eF}} \end{array}} \right]\end{array}$$

where *V*_*pM*_, *V*_*pF*_ are the phenotypic (*p*) variances of the males (*M*) and females (*F*), respectively, and $Cov_{pMF}$ is the phenotypic covariance between the sexes. The phenotypic covariance matrix is decomposed into autosomal additive (*a*) and dominance (*d*), sex-linked additive (*x*), and environmental (*e*) components, where the cross-sex residual covariance is fixed to zero because this parameter is not estimable when traits are only ever expressed in separate female or male environments (Wolak et al. 2015).

Convergence was obtained for only three traits and in all of these the sex-linked additive covariance between the sexes was not statistically different from zero. Therefore, all the models presented in this paper assume a sex-linked additive genetic correlation between the sexes of zero, i.e. $Cov_{xMF} = 0$.

$$\begin{array}{l}\left[ {\begin{array}{*{20}{c}} {V_{pM}} & {Cov_{pMF}} \\ {Cov_{pMF}} & {V_{pF}} \end{array}} \right] = \left[ {\begin{array}{*{20}{c}} {V_{aM}} & {Cov_{aMF}} \\ {Cov_{aMF}} & {V_{aF}} \end{array}} \right] + \left[ {\begin{array}{*{20}{c}} {V_{dM}} & {Cov_{dMF}} \\ {Cov_{dMF}} & {V_{dF}} \end{array}} \right]\\ \qquad\qquad\qquad\qquad\qquad\,+\,\left[ {\begin{array}{*{20}{c}} {V_{xM}} & 0 \\ 0 & {V_{xF}} \end{array}} \right] + \left[ {\begin{array}{*{20}{c}} {V_{eM}} & 0 \\ 0 & {V_{eF}} \end{array}} \right]\end{array}$$

An important consequence of the very small sex-linked genetic variance in males is that the type of dosage compensation, if any, should not matter. We ran the analyses using a no-dosage compensation model (“ngdc” option in R package nadiv; Wolak 2012, 2013) and a full dosage compensation model (“hedo” option in nadiv). As expected, differences between estimates of male sex-linked genetic variance were less than 10^−8^. The results for the variance components as proportions of the total phenotypic variance were identical (i.e., independent of the dosage compensation model used) and thus we report only one set of values.

The usual statistical analysis of linear regression is potentially biased by collinearity, the influence of outliers, excessive leverage and covariance among predictor variables. These factors are hard to evaluate with just 12 data points. To circumvent such problems, we adopted a randomization approach (Roff 2006). The general procedure was as follows: first, we ran the usual linear regression analysis and stored the *F* value, which we designate as *F*_*obs*_. Next, we randomized the abs(SDI) estimates and reran the regression analysis, storing the *F* value as *F*_*1*_. This randomized procedure was repeated 10,000 times. The null hypothesis of no covariation was tested by computing the number of times the *F* value from the randomized data set exceeded *F*_*obs*_, which we denote as $N_{F_i > F_{obs}}$. Probability was estimated in the usual fashion for a randomized protocol as $P_r = \left( {N_{F_i > F_{obs}} + 1} \right)/10,001$, where the extra 1 represents the observed *F* value. For a one-tailed test the randomization approach was modified in that to be included in $N_{F_i > F_{obs}}$ the slope of the regression must also be in the predicted direction. For comparison, we provide the regression estimates for the regression using the observed data set, the associated probability, *P*_obs_, and the probability from the randomization method, *P*_*r*_.

The full genetic model described above (i.e., additive, dominance and sex-linked variance components) was compared to the constants-only model (i.e., with no genetic effects) using the log-likelihood ratio test (Wilson et al. 2010). Convergence was obtained for all morphological traits and in all cases the full model accounted for significantly more variance than the constants-only model and this difference was highly significant (*P* < 10^−6^).

## Results

### Parameter estimates

**Fig. 2:**
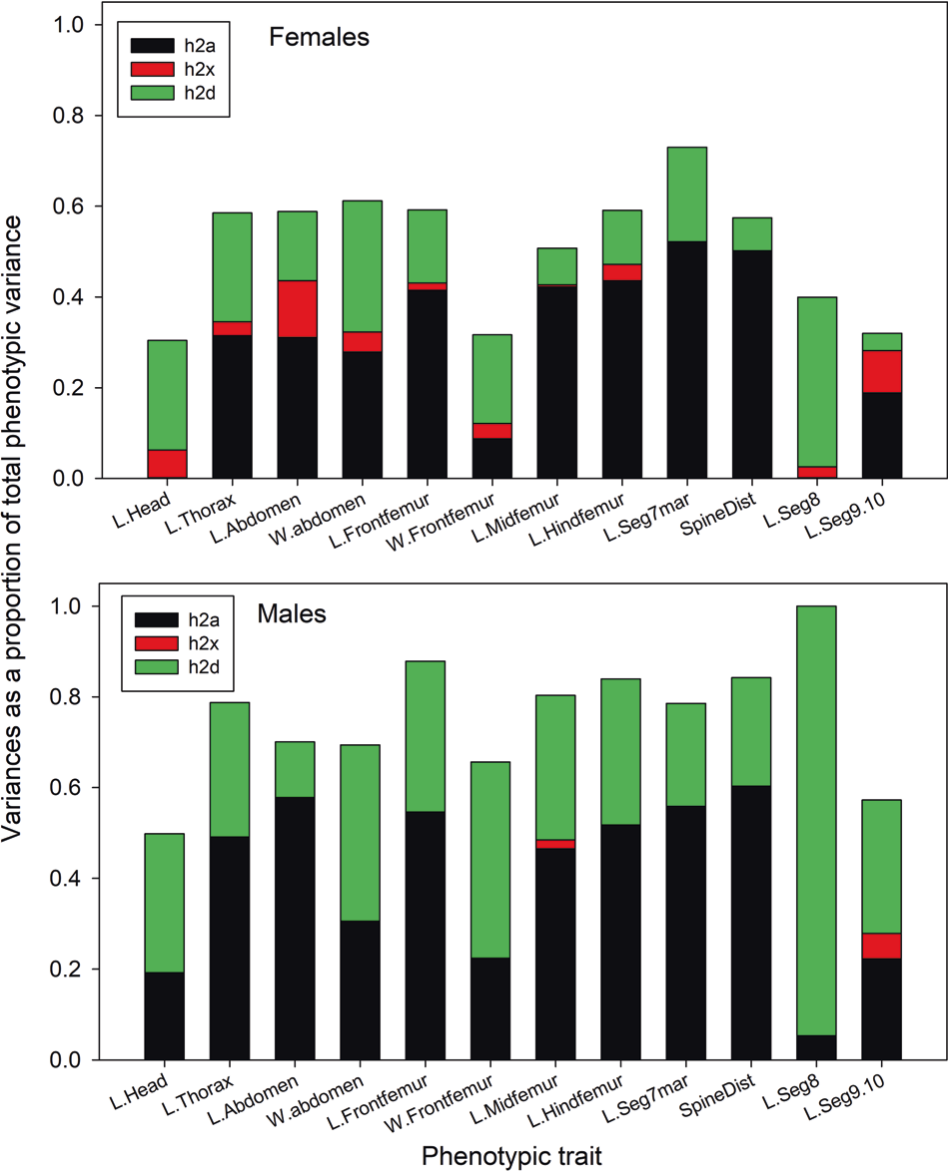
Stacked bar diagrams showing the variance proportions for the 12 measurement traits. Top panel shows females and bottom panel shows males. Colors indicate the autosomal additive (black), sex-linked (red) and autosomal dominance (green) variances as proportions of the total phenotypic variance.

For comparisons with previous studies, the overall heritabilities ($h_A^2$) including both autosomal and sex-linked additive effects were estimated as (*V*_*a*_ + *V*_*x*_)*/V*_*p*_. They averaged 0.40 (SD 0.19) in males and 0.35 (SD 0.15) in females and were significantly greater than zero in both sexes for all traits except L.Seg8 in males (Supplementary Tables S5 and S6). These estimates are very similar to the proportions of autosomal additive variance (autosomal heritabilities, $h_a^2 = V_a/V_p$) estimated from the full model, which averaged 0.40 (SD 0.19) in males and 0.29 (SD 0.18) in females (Fig. 2). This reflects the low proportions of sex-linked variance detected in both sexes, but particularly in males (see below). Of the 24 estimates of $h_a^2$, 19 were significantly greater than zero (Supplementary Tables S5 and S6). For three traits, L.head, W.Frontfemur and L.Seg9.10, $h_a^2$ was statistically significant only in males, and it was not significant in either sex for L.Seg8.

**Fig. 3:**
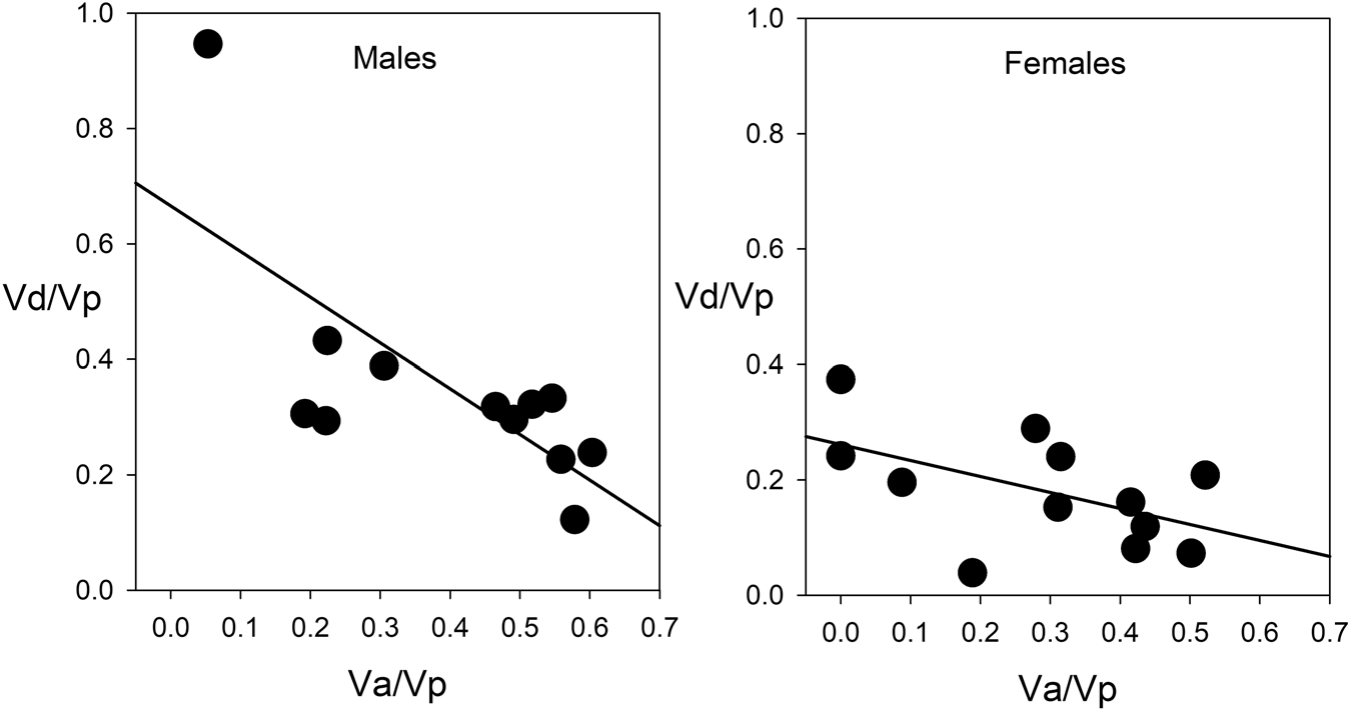
Scatterplot showing the covariance relationship between the autosomal and dominance genetic variance proportions for the 12 traits. Values for males are shown on the left, females on the right. The solid line shows the fitted regression line.

For most traits, the proportion of dominance variance ($h_d^2 = V_d/V_p$) was of similar magnitude to $h_a^2$, particularly in males (males: mean 0.35, SD 0.20; females mean 0.18, SD 0.10, Figs. 2 and 3, Supplementary Table S5). It was significantly greater than zero for 10 traits in males and 5 traits in females. The length of segment 8, which lacked significant autosomal heritability in either sex, had the highest proportion of dominance variance in both sexes (0.95 and 0.37 in males and females, respectively). Head length, which lacked significant autosomal heritability in females, also had relatively high and significant dominance variance.

Our breeding design minimizes sampling correlations between *V*_*a*_ and *V*_*d*_ (Meyer 2008). Nevertheless, we found a negative correlation between $h_a^2$ and $h_d^2$: traits with relatively low proportions of autosomal additive variance tended to have relatively high proportions of dominance variance, especially in males (Fig. 3; *r*_10_ = −0.725, *P* = 0.008 for males, and *r*_10_ = −0.526, *P* = 0.079 for females). This negative correlation could arise in part because of the mathematical relationship between these two proportions, both being derived by division by *V*_*p*_: i.e., *V*_*a*_/*V*_p_ = 1 − *V*_*d*_/*V*_*p*_ *−* *V*_*x*_/*V*_*p*_ − *V*_*e*_/*V*_*p*_. However, this is likely only if $h_a^2$ and $h_d^2$ make up a large portion of *V*_*p*_ and thus are mathematically constrained to covary. In our data, the sum of these two variances comprised an average of only 0.75 and 0.47 of the phenotypic variances in males and females respectively (Supplementary Table S5). Thus, there is plenty of room for the proportions of additive and dominance variance to vary independently of one another. Large values of *V*_*d*_ do not arise simply because of small values in *V*_*a*_ but represent a true pattern in the variance structure within this suite of traits. Thus, our results indicate that dominance variance contributes significantly to genetic variance in our suite of traits and this is particularly true for traits that have relatively low proportions of autosomal additive variance.

As expected from the analyses of each sex separately, the estimated proportions of sex-linked variance ($h_x^2 = V_x/V_p$) were much lower than the other two components, with means of only 0.007 (SD 0.018) in males and 0.039 (SD 0.039) in females (Fig. 2 and Supplementary Table S5). Males had significantly lower proportions of the sex-linked variance than females (paired *t*-test, *t*_*11*_ = 3.079, *P* = 0.010), and none of the estimates were individually significant in males. In females, only L.Head and L.seg8 were significant. It is of note that these are the traits that had non-significant autosomal heritability but large and significant proportions of dominance variance.

### Answering question 1: Are sexual dimorphisms associated with lower additive inter-sex genetic correlations?

**Fig. 4:**
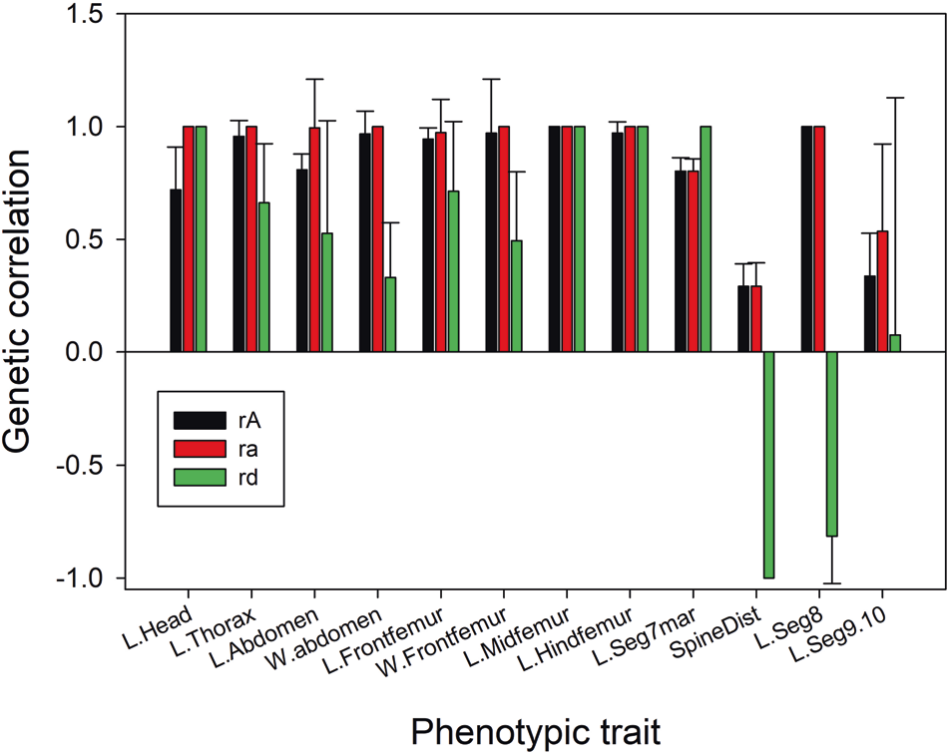
Bar graph of the three types of genetic correlation for the 12 measured traits. Error bars show +1 standard error. Standard errors cannot be calculated for estimates of *r* at boundary conditions (+1.0 or −1.0).

**Fig. 5:**
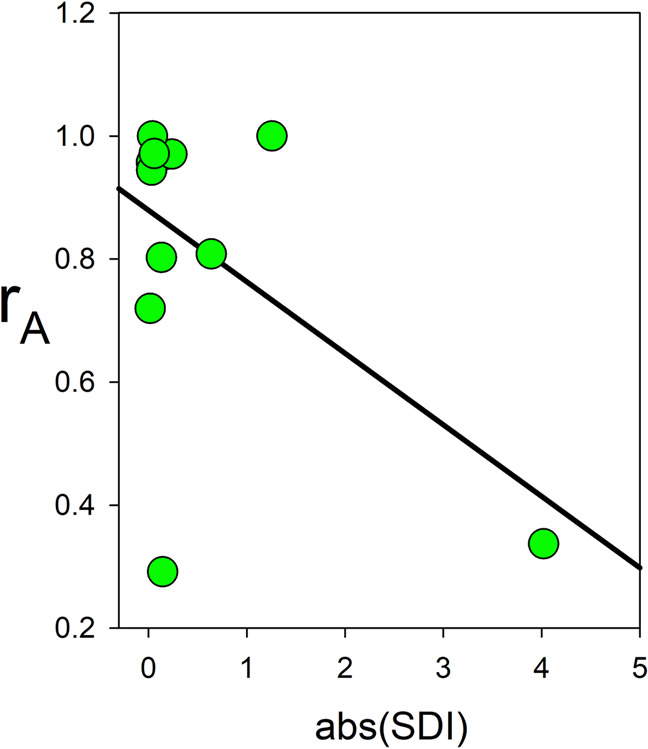
Scatterplot showing the relationship between the overall additive inter-sex genetic correlation (*r*_*A*_) and abs(SDI). Line shows the linear regression of *r*_*A*_ on abs(SDI).

We first looked for this relationship using the overall additive inter-sex correlations, *r*_*A*_ (Fig. 4; numerical data shown in Supplementary Table S5). As predicted, *r*_*A*_ correlated negatively with abs(SDI), but this was statistically significant only in the standard analysis (*r*_*10*_ = −0.53, *P*_*obs*_ = 0.037, *P*_*r*_ = 0.126, one-tailed test as the predicted relationship is negative, Fig. 5). The significance from the standard analysis clearly arises from the high leverage of a single datum (L.Seg.9.10). The correlation using the autosomal additive correlation, *r*_*a*_, was also negative, but it was not significant in either analysis (*r*_*10*_ = −0.41, *P*_*obs*_ = 0.092, *P*_*r*_ = 0.112, one-tailed tests). As expected, *r*_*A*_ tends to be lower than *r*_*a*_ (paired *t*_11_ = 2.490, *P* = 0.015, one-tailed test), and this difference is proportional to $h_x^2$ (*r*_*a*_ – *r*_*A*_ = 0.0173 + 0.3172 $h_x^2$, *F*_1,10_ = 17.55, *P* = 0.0019). These results are consistent with the expectation that sex-linked additive variance lowers *r*_*A*_.

We asked if the relationship between *r*_*a*_ and abs(SDI) might be confounded by the effects of the dominance inter-sex correlation, *r*_*d*_, because the two correlations were significantly correlated with each other (*r* = 0.59, *P* = 0.043). Linear regression of abs(SDI) on *r*_*d*_ alone was not significant by either the standard or randomization analyses (*P*_*obs*_ = 0.291, *P*_*r*_ = 0.250), and, although multiple regression with both correlations found significant effects of all components (abs(SDI) = 4.81–4.12*r*_*a*_ + 4.76*r*_*d*_–5.70*r*_*a*_*r*_*d*_; *P*_*obs*_ = 0.024; all coefficients with *P* < 0.02), the randomization was not significant (*P*_*r*_ = 0.158). Thus inclusion of *r*_*d*_ did not reveal a more robust relationship between abs(SDI) and *r*_*a*_.

### Answering question 2: Are sexual dimorphisms associated with higher proportions of sex-linked variance?

To address this question, we first did simple linear regression of abs(SDI) on $h_x^2$ for females. The relationship was positive, but significant only for the parametric analysis (abs(SDI) = −0.02 + 14.78 $h_x^2$; *t*_10_ = 1.808, *P* = 0.049, *P*_*r*_ = 0.097, one-tailed because the predicted relationship is positive).

To determine if this relationship is influenced by the other two variance components, we ran stepwise multiple regression of abs(SDI) on the three variance components. The variances tended to covary negatively but were not significantly correlated with each other:$h_x^2$ vs $h_d^2$, *r* = −0.10; $h_a^2$ vs $h_x^2$, *r* = −0.40; $h_a^2$ vs $h_d^2$, *r* = −0.53; *P* = 0.758, 0.194, 0.079, respectively. Thus, collinearity was not likely to bias the regression. The final model consisted of all possible components, ${{{\mathrm{abs}}}}\left( {{{{\mathrm{SDI}}}}} \right) = - 3.8 + 20.5h_d^2 + 8.3h_a^2 + 133.2h_x^2 - 41.8h_d^2h_a^2 - 626.3h_d^2h_x^2 - 300.7h_a^2h_x^2 + 1321.8h_d^2h_a^2h_x^2$ (*F*_7,4_ = 44.9, *P*_*obs*_ = 0.001, *P*_*r*_ = 0.024, *P* values for all coefficients <0.015). To more fully understand these complex relationships, we did randomization regressions for each of the variance components alone and for the three pairwise combinations. Of these, only the regression with $h_x^2,\,h_d^2$ was significant (abs(SDI) = −1.15 + 9.43 $h_d^2$ + 57.62 $h_x^2$−334.98 $h_x^2h_d^2$; *P*_*obs*_ = 0.003, *P* values for all coefficients <0.05, *P*_*r*_ = 0.021). Thus, in spite of the complexity of the full model, the pairwise analyses suggest that the components likely contributing most to the covariation with abs(SDI) are $h_x^2$ and $h_d^2$.

We were unable to duplicate these analyses in males because of the lack of variation in $h_x^2$ (Fig. 2 and Supplementary Table S5). However, multiple regression using only $h_a^2$, and $h_d^2$ was not significant (*P*_*obs*_ = 0.386, *P*_*r*_ = 0.276).

### Answering question 3: Are sexual dimorphisms associated with differences between sexes in autosomal additive and dominance genetic variances?

Looking first at autosomal additive genetic variance, we find evidence of both similarities and differences between the sexes. The estimates of *r*_*a*_ were ≥0.97 for all traits except L.Seg7marg, SpineDist and L.Seg9.10 (Fig. 4 and Supplementary Table S5), and the correlation of $h_a^2$ between males and females was highly significant (*r* = 0.917, *F*_1,10_ = 52.58, *P* < 0.001). These results suggest that the autosomal additive effects covary between sexes both within and between traits. However, covariance does not mean that the additive variances do not differ between the sexes. To the contrary, $h_a^2$ was consistently higher in males than in females (Fig. 2 and Supplementary Table S5; paired *t*-test, *t*_11_ = 4.797, *P* < 0.001), and this was due to males having slightly larger *V*_*a*_ (mean male–female = 0.126, paired *t*-test: *t*_11_ = 2.018*, P* = 0.069) rather than smaller *V*_*p*_ (mean male–female difference = 0.144, paired *t*-test: *t*_11_ = 1.213, *P* = 0.251). In addition, *r*_*a*_ was significantly less than 1.0 for L.Seg7marg and SpineDist, and was not significantly greater than zero for L.Seg9.10. Both the overall higher $h_a^2$ in males and the low *r*_*a*_ for three traits indicate the presence of sex-specific patterns of autosomal additive variance.

To determine if these differences between the sexes are associated with sexual dimorphism we expressed the magnitude of the difference in variances as the ratio of the larger variance to the smaller variance for each of the 12 traits, and then estimated the correlation between abs(SDI) and the variance ratios. The correlation estimate was positive but not significant (*P*_*obs*_ = 0.753, *P*_*r*_ = 0.227). Thus, within our suite of traits, the magnitude of the difference between sexes in autosomal additive variation is not correlated with the magnitude of sexual dimorphism.

We analyzed the autosomal dominance variances following the same protocol. In a pattern similar to the additive variances, $h_d^2$ was significantly correlated between sexes across the 12 traits (*r* = 0.66, *F*_1,10_ = 7.746, *P* = 0.019) and was significantly larger in males than in females overall (paired *t*-test, *t*_11_ = 3.793, *P* = 0.003). As with $h_a^2$, this difference between the sexes was due to the dominance variance being higher in males (mean male–female = 0.16, paired *t*-test: *t*_11_ = 1.904*, P* = 0.083) rather than the phenotypic variance being lower. However, unlike the additive inter-sex correlations, the inter-sex dominance correlations (*r*_*d*_) were quite variable in both magnitude and sign (Fig. 4 and Supplementary Table S5). On average, *r*_*d*_ was positive (mean = 0.42, SD = 0.68; one-sample *t*-test, *t*_11_ = 2.104, *P* = 0.059), but in two cases (L.Seg8 and SpineDist), it was strongly and significantly negative. Of the 10 traits with positive estimates, four were not significantly greater than zero (L.Abdomen, W.Abdomen, W.Frontfemur and L.Seg9.10). These results indicate that dominance variances differ between sexes for some traits, and that these differences can be extreme (as in the two cases of strong negative *r*_*d*_).

In contrast to the additive variances, the correlation between the ratio of dominance variances (larger/smaller) and abs(SDI) was significant and strongly positive (*r* = 0.961, *F*_1,10_ = 125.8, *P*_*obs*_ < 0.001, *P*_*r*_ = 0.003). This indicates a very strong relationship between the magnitude of the difference between sexes in dominance variance and the magnitude of sexual dimorphism.

Taken together, these results show that sexual dimorphism is associated with sex-specific patterns of autosomal genetic variance and that this association is due primarily (perhaps entirely) to the contribution of strong, sex-specific patterns of dominance variance. Thus, for our data, the answer is affirmative for question 3.

## Discussion

In this paper, we applied the method described by Fairbairn and Roff (2006) to estimate the autosomal and sex-linked genetic (co)variances for a suite of 12 size traits in the water strider, *A. remigis*. Our estimates revealed previously unknown aspects of the quantitative genetic architecture of these traits. In addition, because the magnitude of sexual dimorphism differed greatly among traits, we were able to use comparisons among traits to address three key questions about the quantitative genetic architecture of sexual size dimorphism in this species.

### General conclusions concerning the genetic architecture of size traits in *A. remigis*

As predicted, given our choice of traits, the matrix of genetic correlations among traits demonstrates considerable genetic independence: all of the between-trait additive genetic correlations were less than |1|, with averages in both sexes of less than 0.5. Although webs (matrices) of genetic correlations among traits may be more constraining than pairs of within-trait correlations might suggest (Gosden et al. 2012; Wyman et al. 2013), as can differences in cross-trait correlations between sexes (**B** matrix; Wyman et al. 2013), the relatively low correlations that we observed nevertheless suggest the potential for continued evolutionary divergence of trait sizes should selection continue to favor such change (Preziosi and Fairbairn 2000; Ferguson and Fairbairn 2000; Bertin and Fairbairn 2005).

Both the overall and the autosomal heritabilities ($h_A^2$ and $h_a^2$) were generally moderate and significant, as is typical for morphological traits (Mousseau and Roff 1987). There were exceptions to this pattern: the length of segment 8 had very low values for both heritabilities, with only $h_A^2$ in males significantly greater than zero. Three other traits, head width, width of the front femur and length of segments 9 and 10, had non-significant autosomal heritabilities in females. However, low heritabilities did not mean lack of genetic variance, because the proportion of dominance variance tended to be high when heritabilities were low. Thus, traits with relatively low heritability tended to have significant reserves of genetic variance in the form of dominance variance.

The proportions of dominance variance ($h_d^2$) were generally significant, with means of 18% for females and 35% for males. These proportions approach the proportions of autosomal additive variance and are higher than the average of 14% estimated by Wolak and Keller (2014) in their review of 559 estimates from 89 papers. The estimates of dominance variance may be inflated by maternal effects because, as noted previously, the variance component extracted using our methodology includes both dominance and maternal effects. However, the latter are likely to be small for adult traits (Supplementary Fig. S1), accounting for only an average of 3% of the phenotypic variance. Even allowing for this, the dominance proportions that we have found would still exceed the average and, for males at least, would approach the proportions of autosomal additive variance.

Relatively high levels of dominance variance could facilitate rapid response to selection even in traits with low heritability if allele frequencies were to shift due to drift or strong selection, thereby converting some dominance variance into additive variance (Robertson 1952; Willis and Orr 1993; Taft and Roff 2012). The potential for such shifts is likely to be high in *Aquarius remigis*, given the population extinction-recolonization dynamics with very small initial populations and the small effective sizes (*N*_*e*_ = 170) characteristic of most populations (Fairbairn 1985a, b; Preziosi and Fairbairn 1992). This mechanism of generating new additive variation may facilitate the adaptive divergence in size traits documented among populations of *A. remigis* experiencing different social or environmental conditions, even on small geographic scales (e.g., Blanckenhorn 1991; Fairbairn and Preziosi 1994; Blanckenhorn and Fairbairn 1995).

In contrast to the other variance components, the proportions of sex-linked variance ($h_x^2$) were generally low, averaging only 0.7% in males and 3.9% in females, with maxima of only 6% and 13%, respectively. The low proportions of X-linked variance suggest that X-linked effects are not disproportionally large relative to the size of the X chromosome, which comprises approximately 7% of the diploid chromosome complement of males and 14% of females (Fairbairn et al. 2016). Of course, we do not know to what extent, if any, chromosome size comparisons reflect the relative proportions of genes and *nc*DNA regulatory sequences on the X chromosome versus autosomes. Nevertheless, this crude comparison suggests that X-linked genes are not contributing more to the phenotypic variance in size traits than would be expected given the relative size of the X chromosome, even for our most sexually dimorphic traits.

### Comparisons among traits: sexual dimorphism and inter-sex genetic correlations

Our first question was whether sexual dimorphism is associated with a reduction in the overall additive inter-sex genetic correlation. Although our results do show this trend for both *r*_*A*_ and *r*_*a*_, it was statistically significant only for *r*_*A*_ and then only with the parametric test. This contrasts with the strong negative correlation between the magnitude of sexual dimorphism and the inter-sex genetic correlation found in Fairbairn (2007). The earlier study included only four somatic measures and one genital measure (total genital length). By comparison, we measured 10 somatic and two genital components. Furthermore, we were able to separate additive and dominance covariances, whereas the earlier study used full-sib estimates of *r*_*A*_, which could have included some covariance due to dominance effects. We found a positive correlation between *r*_*a*_ and *r*_*d*_, suggesting that the inclusion of dominance covariance might have inflated the previous full-sib estimate of *r*_*A*._ We cannot say if these differences between studies fully account for the weaker correlation found in our analysis. Perhaps the base populations used for the two studies truly differ in the strength of this relationship. Whatever the cause, the lack of significance of the correlation in our randomization analysis means that the answer to question 1 remains uncertain with respect to *A. remigis*.

### Comparisons among traits: sexual dimorphism and X-linked variance

Our second question was whether or not sexual dimorphism is associated with increased proportions of X-linked variance (recall that *A. remigis* has no Y chromosome, so sex-linkage = X-linkage in our analyses). We tested for this relationship only in females because the X-linked variance was negligible for most traits in males. The estimated correlation between abs(SDI) and $h_x^2$ was positive and was significant with parametric analysis but not with randomization. However, this relationship was complicated by significant covariation of abs(SDI) with the effects of the other variance components: abs(SDI) covaried with $h_x^2$, $h_a^2$, $h_d^2$, and their interactions. Analysis of individual components suggested that $h_x^2$ and $h_d^2$ contributed most significantly to the overall covariance.

These findings point again to the importance of developing a theory for the genetic basis and evolution of sexual dimorphism that explicitly includes dominance variance in addition to autosomal additive and X-linked variances. A possible interpretation of interactions seen here and in the analysis of genetic correlations is that the genetic pathways by which sexual dimorphism evolves may vary among traits. For example, the conversion of autosomal additive variance to dominance variance or to X-linked variance provides alternative mechanisms for reducing genetic constraints on the evolution of sexual dimorphism, and the relative importance of these two mechanisms may vary from trait to trait as well as between sexes for any given trait. Our results suggest greater importance of dominance in males relative to females, whereas the reverse is true for X-linkage. A more detailed mapping of the patterns of covariation signaled by our significant interaction terms could shed light on this hypothesis but must await studies examining a greater number of traits.

### Comparisons among traits: sexual dimorphism and differences between sexes in autosomal additive and dominance genetic variances

Our final question asked if sexual dimorphism is associated with differences between sexes in autosomal additive and dominance variances. We found consistent differences between the sexes in both additive and dominance autosomal variance components, with males having significantly higher proportions of both. For additive variances, we found no indication of the pattern of differences between sexes suggested by the interspecific comparative study of Wyman and Rowe (2014). They found that heritabilities were significantly larger in males than in females for the traits under sexual selection (termed “Reproduction”), whereas the opposite was true for non-sexually selected traits. We did not find this distinction in our data for *A. remigis*. Our suite of 12 traits includes traits experiencing a wide range of sexual selection intensity: previous multivariate analyses of selection indicate that sexual selection primarily favors longer genitalia in males and this selection is often strong (Preziosi and Fairbairn 1997, 2000; Preziosi et al. 1996; Ferguson and Fairbairn 2000). In contrast, somatic size traits tend to be much more strongly influenced by selection acting through differential survival and fecundity (*op.cit*.). Nevertheless, we found that the proportion of autosomal additive variance ($h_a^2$) was consistently higher in males than in females across all traits. Using overall heritabilities ($h_A^2$), which are more comparable to the estimates in Wyman and Rowe (2014), gave the same results (Supplementary Table S5; paired *t*-test, *t*_11_ = 4.536, *P* < 0.001): overall heritabilities are larger in males for all traits except abdomen width (females larger by only 0.01) and the length of segments 9 and 10 (sexes equal). Thus, we find no indication of the distinction noted by Wyman and Rowe between sexually selected and non-sexually selected traits, and no indication that any of the traits we used have higher heritabilities in females.

The key prediction for answering question 3 is that the magnitude of these sex differences in autosomal variances should be positively correlated with the magnitude of sexual dimorphism. This prediction was not supported for autosomal additive variances but was strongly supported for autosomal dominance variances. Thus, for our traits, sexual dimorphism is positively associated with the magnitude of the difference between the sexes in the proportion of autosomal genetic variance, but this is true only for the dominance variance component. This pattern of association between sexual dimorphism and sex-specific patterns of dominance variance aligns with mounting empirical evidence for sex-specific dominance associations in traits under sexually antagonistic selection (e.g., Barson et al. 2015; Grieshop and Arnqvist 2018; Kaufmann et al. 2021).

## General conclusions

The clearest conclusion that arises from our comparative analysis of traits in *A. remigis* is that differences in trait size between males and females are strongly correlated with sex differences in the proportion of autosomal dominance variance. Furthermore, the proportion of the phenotypic variance in trait size that is attributed to dominance variance is high, rivaling the proportion attributable to additive variance. In contrast, the proportion of sex-linked variance is low and, at best, weakly associated with the magnitude of sexual dimorphism only in females. Autosomal additive variance, although comprising the largest of the variance components, shows the least association with sexual dimorphism, the only hint of a possible relationship being the slight, non-significant negative association between *r*_*a*_ and abs(SDI). Thus, we found a clear positive answer only to the last of the three questions that framed our analyses: sexual dimorphisms for body size traits in *A. remigis* are associated with sex differences in autosomal genetic variance and this is due to large differences in the proportions of autosomal dominance variance. Unfortunately, our data do not provide clear answers to questions 1 and 2. In both cases, we found weak, non-significant trends in the expected directions, so we cannot say with assurance that the predicted relationships exist, nor can we say that they do not.

One cause of the lack of resolution of these questions was the pattern of strong interactions between the sex-linked and dominance variance components, suggesting that the primary genetic pathways by which sexual dimorphism evolves may differ among traits within a given species. A comparison of our results with the results from Kaufmann et al. (2021) suggests that even greater differences are likely between species with differing sex-determining genetic systems. Their analysis of body size (weight) in the seed beetle, *Callosobruchus maculatus*, revealed significantly more autosomal additive and dominance variance in females than males, the opposite of the pattern seen in *A. remigis* and, unlike in *A. remigis*, the proportions of dominance variance were very low and significant only in females. Even more striking is the difference between species in the pattern of sex-linked variance. Water striders lack a Y chromosome (Fairbairn et al. 2016) and hence have no Y-linked genetic variance. In contrast, seed beetles have a Y chromosome and Y-linked additive variance accounted for more than half of the total additive variance detected in male *C. maculatus*. Most significantly, this Y-linked additive variance accounted for most of the response of selected lines of *C. maculatus* to sexually antagonistic selection favoring increased sexual dimorphism. Thus, Y-linkage facilitated the evolution of sexual dimorphism in *C. maculatus* while autosomal dominance variance did not, a marked contrast to the pattern we found in our comparison of traits in *A. remigis*. Given these differences, it will be interesting to see what patterns may emerge as similar analyses are applied to other species, particularly those with differing sex-determining systems.

Overall, our findings of genetic differences between the sexes are consistent with the hypothesis that the evolution of sexual differences in *Aquarius remigis* has been facilitated by the evolution of sex-specific autosomal dominance effects. These results indicate that dominance effects may be more significant than previously appreciated and that their omission from most quantitative genetic studies of sexually dimorphic traits is problematic. Our results also reveal complex interactions among the autosomal additive, autosomal dominance, and sex-linked components of genetic (co)variation, indicating that the genetic pathways by which sexual dimorphism evolves may vary among traits, as well as between sexes. These results contrast sharply with those of Kaufmann et al. (2021), and taken together, the two studies highlight the importance of including both dominance and sex-linked components of genetic (co)variation in future studies of the evolution and genetic architecture of sexual dimorphism.

## Supplementary information

Supplementary Information

## Data Availability

All data for the pedigree experiment have been archived in Dryad (10.6086/D1P09P).
