# Supplementary material for: Tests for associations between sexual dimorphism and patterns of quantitative genetic variation in the water strider, Aquarius remigis
Source: Heredity (Edinb). 2023 May 29;131(2):109–18. doi: 10.1038/s41437-023-00626-5 (PMC10382563; doi:10.1038/s41437-023-00626-5)
Supplement: Supplementary file 1 — Supplementary Information [file 41437_2023_626_MOESM1_ESM.pdf]

Tests for Association between Sexual Dimorphism and Patterns of Quantitative Genetic Variation in the Water Strider, *Aquarius remigis*

Authors: Fairbairn DJ, Roff DA, Wolak ME

SUPPLEMENTARY INFORMATION

The following supplementary information is provided:

A. Prior estimates of sex-linked variances.
B. Photographs of male and female *Aquarius remigis* showing the measurements used in the study.
C. Dominance and maternal variances.
D. Characteristics of the founding populations.
E. Details of the laboratory rearing regime.
F. Details of the experimental protocols for the pedigree rearing experiment.
G. Details of the measurement protocols.
H. Results of our analysis of genetic correlations among traits with each sex.
I. Genetic variances (as proportions of phenotypic variance) and between-sex genetic correlations for the 12 measured traits.
J. A list of references used in the Supplementary Information.

A. Sex-linked variance estimates

Table S1: Studies reporting sex-linked genetic variance. Columns show the percentages of phenotypic variance ascribed to autosomal additive variance (h_a^2 %), X- or Z-linked variance (h_x^2 %), and autosomal dominance variance (h_d^2%). Data obtained using a search of web of science with keywords "sex-linked variance", "dominance variance", "heritability" and variants of these. These papers were themselves searched for additional relevant references. Simple means were calculated using all the reported estimates in each paper.

Species	Name	Sex[a]	D[b]	h_a^2%			h_x^2%			h_d^2%				Ref
				range	$\bar{x}$	SD	range	$\bar{x}$	SD	range	$\bar{x}$	SD	n	
D. melanogaster	Fruit fly	M	2	23-56	38	11	0-30	12	10	Excluded			15	1
		F	2	25-68	38	12	0-26	9	9	Excluded			15	
D. melanogaster	Fruit fly	M	2	23-59	37	11	0-30	17	8	Excluded			13	2
		F	2	25-55	39	10	2-17	10	4	Excluded			13	
D. melanogaster	Fruit fly	M	5	30-77	57	14	0-34	12	9	Excluded			24	3
		F	5	33-77	56	15	0-57	14	14	Excluded			24	
Taeniopygia guttata	Zebra finch	M	4	na	27	na	na	4	na	Excluded			1	4
		F	4	na	27	na	na	4	na	Excluded			1	
Homo sapiens	Human	M	6	0-74	29	28	17-46	33	10	0-34	15	17	6	5
		F	6	0-89	29	33	0-61	23	25	0-42	17	21	7	
Homo sapiens	Human	M	6	11-34	19	13	0-9	6	5	0-48	27	25	3	6
		F	6	0-19	6	11	5-27	17	11	0-9	3	5	3	
Callosobruchus maculatus	Beetle	M	4	26-41[c]	33		0-12[b]	6		0			1	7
		F	4	57-74[c]	66		0-7[b]	2		0			1	
Tribolium casteneum	Flour beetle	B	1	34-44	39	7	4-5	5	1	Excluded			2	8
D. melanogaster	Fruit fly	F	6	32-42	37	7	7-15	11	6	Excluded			2	9
Lymantria dispar	Gypsy moth	F	1	6-51	26	20	0-0	0	0	Excluded			4	10
Bombyx sp.	Silk moth	B	1	20-45	33	18	3-40	22	26	20-33	27	9	2	11

Parus major	Great tit	B	3	na	23	na	na	5	na	Excluded			1	12
Taeniopygia guttata	Zebra finch	B	4	8-65	34	23	0-7	3	3	Excluded			5	13
Ficedula albicollis	Flycatcher	B	3	2-36	21	15	0-40	12	16	Excluded			5	13
Tyto alba	Barn owl	B	3	na	57	na	na	13	na	Excluded			1	14
Tyto alba	Barn owl	B	3	15-50	30	18	16-50	38	19	Excluded			3	15
Tyto alba	Barn owl	B	3	na	48	na	na	30	na	Excluded				16
Aphelocoma coerulescens	Florida scrub-jay	B	3	0-38	21	19	0-0	0	0	Excluded			3	17
Taeniopygia guttata	Zebra finch	M	4	0-18	9	13	0-4	2	3	Excluded			2	18
Sus scrofa	Pig	B	4	4-11	7	5	0-0	0	0	Excluded			2	19
Papio hamadryas	Baboon	B	4	48-87	58	10	0-18	4	6	Excluded			16	20
Mus musculus	Mouse	B	1	35-41	38	4	0-28	14	20	0-4	2	2	2	21

a: M=male, F=female, B=both

b: Experimental design 1=line-cross; 2=half-sib; 3=wild population, animal model; 4=captive population, animal model; 5=half-sib and offspring on parent; 6=Maximum likelihood method; 7=Line-cross

c: 95% confidence

References: 1.Cowley and Atchley (1988), 2. Cowley et al (1986), 3. Mezey and Houle (2005), 4. Evans et al (2014), 5. Pan et al. (2007), 6. Kosova et al. (2010), 7. Kaufmann et al. (2021), 8. Carbonell et al (1985), 9. Griffin et al. (2016), 10. Keena et al. (2007), 11. Zhu and Weir (1996), 12. Evans et al (2014), 13. Husby et al (2013), 14. Roulin et al. (2010), 15. Roulin and Jensen (2015), 16. Larsen et al. (2014), 17. Tringali et al. (2015), 18. Husby et al (2013), 19. Wittenburg et al. (2011), 20. Willmore et al. (2009), 21. Zhu and Weir (1996).

Figure S1. Plots of male versus female genetic (autosomal and X-linked) variances as a percentage of the phenotypic variance. The diagonal is the 1:1 line. Note that male and female autosomal variances covary whereas X-linked variances show no such pattern. Different colors show the individual data for the relevant studies shown in Table S1. Cowley et al. (1986, black), Mezey and Houle (2005, red), Evans et al. (2014, blue), Cowley and Atchley (1988, green), Pan et al. (2007, yellow), Kosova et al. (2010, purple), Kaufman et al. (2021, grey)

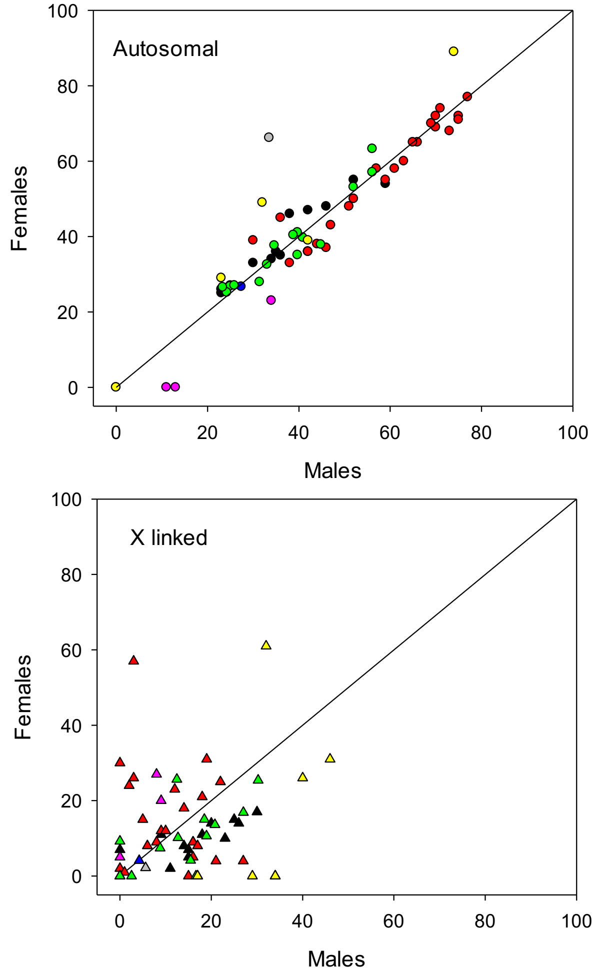

B. Photographs of male and female *Aquarius remigis* showing the measurements used in the study.

Figure S2: Photographs of a male (left) and a female (right) *A. remigis* in ventral view, with arrows indicating the somatic measurements used in this study. Femoral measurements are shown only for the female, but the landmarks are identical in males. Full names and descriptions of each measurement are given in the main paper.

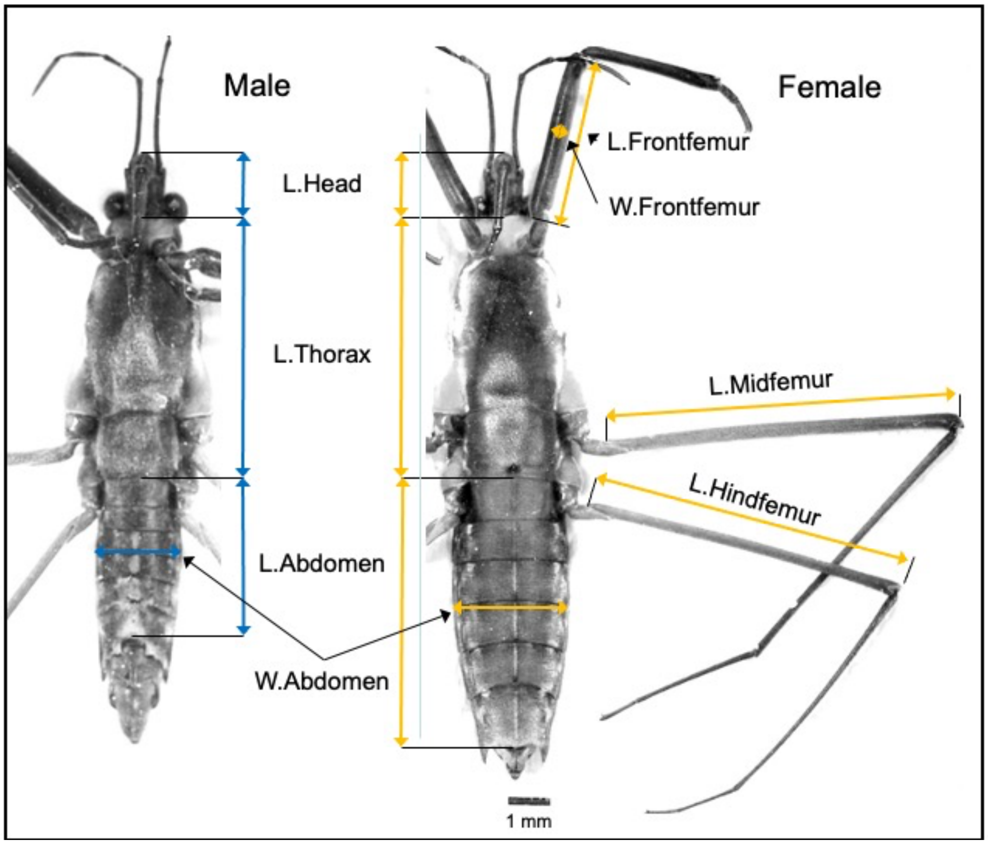

Figure S3: Photographs of the terminal abdominal and genital segments of a male (left) and a female (right) *A. remigis* showing the measurements used in this study. Full names and descriptions of each measurement are given in the main text.

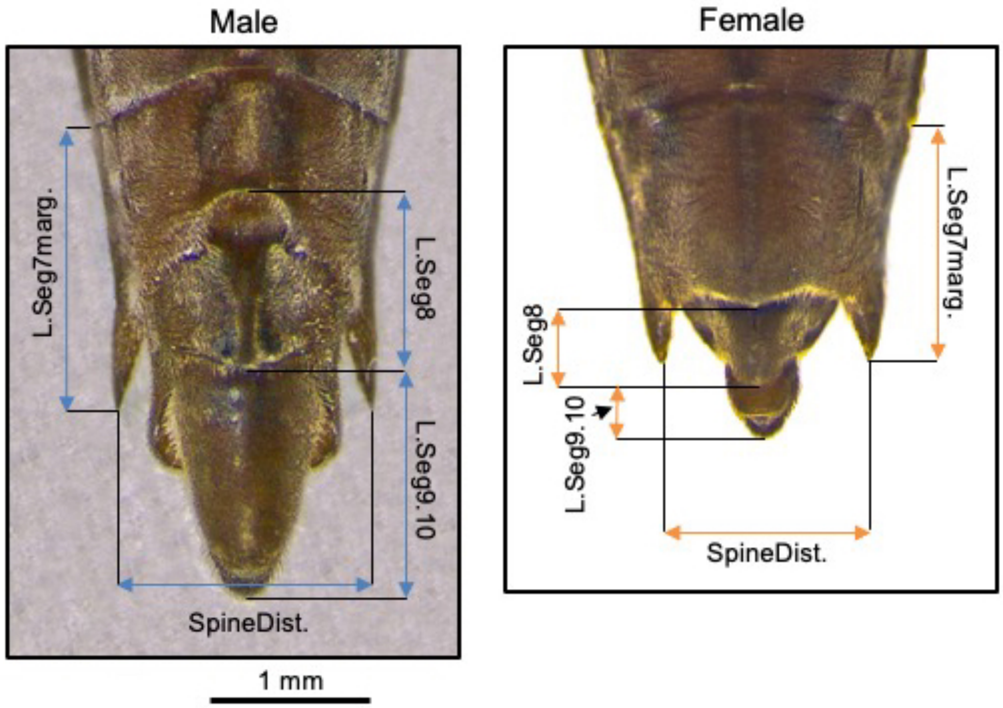

C. Dominance and maternal variances

Most methods cannot estimate both autosomal dominance and additive X-linked which, given question 3, makes such approaches inappropriate for testing hypotheses concerning sexual dimorphisms. Unmanipulated pedigree designs can, in principle, estimate both autosomal additive and autosomal dominance genetic variances. However, in practice, the number and variety of relationships that arise are generally not sufficient for models with dominance to converge or provide accurate estimates (Wolak and Keller 2014). Thus, dominance variances have been typically omitted in studies of sex-linked variances (see Table S1).

Maternal effects are also frequently omitted. Maternal effects are most frequently found during juvenile or larval stages but not in adults (Mousseau and Dingle 1991; Fox 1994; Fox and Savalli 1998; Cotter et al. 2004; Roff and Sokolovska 2004; Keena et al. 2007). This effect is shown in Figure S1, where estimates of maternal effects from the studies in Table S1 that reported these have been divided into juvenile versus adult traits. The cumulative frequency distributions clearly differ, with juvenile traits having much greater values than adult traits. This is reflected in the mean values of 17% (SD=14, SE=3) for juvenile traits and only 3% (SD=5, SE=1) for adult traits.

Figure S4: Cumulative frequency plot of maternal variance as a percentage of the total phenotypic variance for juvenile versus adult traits. Data are from those studies given in Table S1 that provide maternal estimates (12 studies, 58 adult traits, 18 juvenile traits) Data sources are indicated in the reference list by "*".

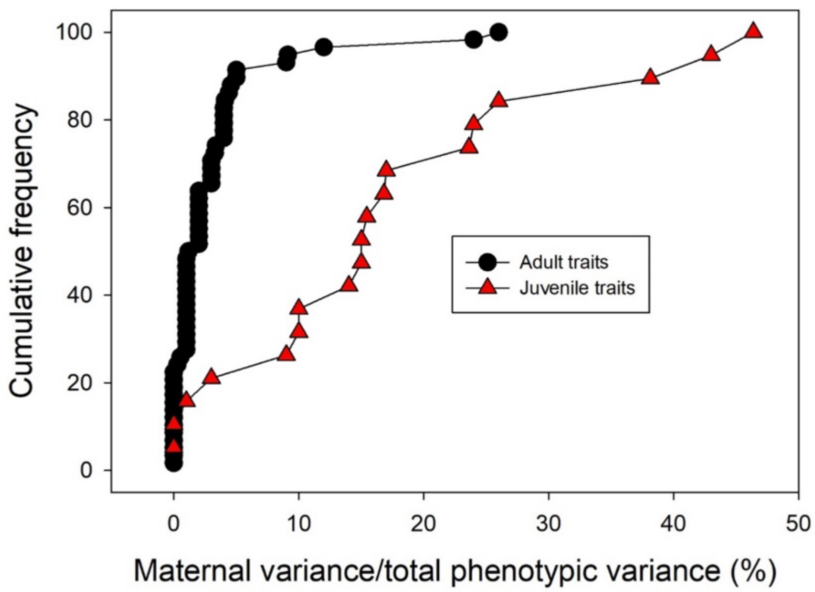

D. Characteristics of the founding populations.

The founders for each replicate were adult *A. remigis* collected from Rattlesnake Creek (latitude 34.459683°N, longitude 119.692105°W), a small, rocky, permanent stream in Santa Barbara, County, California. The founding animals for replicates 1 and 3 were collected during consecutive peak reproductive seasons (Table S2) and the following day were placed in the experimental protocol. The founders for replicate 2 were collected the intervening October (Table S2) and were maintained in stream tanks until the onset of reproduction (approximately 3 months) before being placed in the experimental protocol.

Table S2: Characteristics of founding samples[1] of adult *A. remigis* for each replicate in the pedigree experiment.

Replicate	Date collected d/m/y	Total number of adults captured	Percent wingless[2]	Number used as founders	
				Males	Females
1	17/06/08	248	87.1%	100	100
2	23/10/08	248	91.5%	78	46
3	15/06/09	267	92.9%	105	105

[1]Collected from Rattlesnake Creek in southwestern California (latitude 34.459683°N, longitude 119.692105°W)
[2]Across North America, populations of *A. remigis* typically contain less than 1% winged adults (Calabrese 1974; Fairbairn 1986; Fairbairn and Desranleau 1987). However, winged individuals are more common in California than in other regions (Calabrese, 1974; Polhemus and Chapman, 1979; Kaitala and Dingle, 1992; Fairbairn and King, 2009). Winged adults differ slightly from wingless forms in both size and shape (Fairbairn, 1992), and to avoid this potentially confounding effect, we used only wingless adults to initiate our breeding populations.

E. Rearing regime for *Aquarius remigis*

All rearing and adult maintenance was done in plastic 'shoe-box' cages (34 x 24 cm) partially filled with water, or in oval-shaped, stream tanks (140 cm x 45 cm) with circulating water, under 14hL:10hD, and at room temperature (laboratory) or 25°C (environmental chambers), as noted. Differences in sample sizes and seasons necessitated minor alterations to the protocol for the different replicates, but the same general protocol was followed for all.

To maintain *A. remigis* nymphs and adults in laboratory culture, the water surface must be kept moving. Survival is poor on stagnant water. A circulating current was maintained in the stream tanks by means of submerged pumps controlled with rheostats. The water was also filtered through aquarium pumps and aerated with aquarium air stones (bubblers). In the smaller, 'shoebox' rearing cages, surface movement was maintained by an aquarium bubbler in each cage. Cages and stream tanks were also provided with floating Styrofoam pieces as resting and oviposition sites. To prevent escapes, all tanks and cages were covered with cheesecloth or screen which is sealed around the edges with double-sided tape.

We used the following feeding protocol, which has been shown to produce maximum survival under our rearing conditions: a minimum of one *D. melanogaster* per day for each first and second instar nymph, supplemented with one medium-sized, nymphal house cricket, *Acheta domestica*, per day for every two third or fourth instar nymphs. Fifth instar nymphs and adults were fed at the rate of one adult *A. domestica* per day for every four individuals plus twice weekly supplements of one *D. melanogaster* per individual. All food items were previously frozen. The old crickets were removed from the water surface daily to prevent lipids from the decomposing bodies from compromising the water surface tension. Similarly, the *Drosophila* carcasses are removed at least twice a week. See Blanckenhorn et al (1995) for more information.

F. Experimental Protocols

To initiate each replicate, the field-caught adults from Rattlesnake Creek (Table S2) were distributed among laboratory stream tanks. The founders for replicates 1 and 3 were evenly distributed among four tanks, with equal sex ratios within tanks and total population sizes of 200 and 210 respectively. Replicate 2 had only two stream tanks with 25 females/42 males in one, and 21 females/36 males in the other (total N = 124). After tank set-up, all eggs laid in the first seven days were discarded. This minimized possible environmental maternal effects on egg quality and increased the probability that eggs harvested from each tank were sired by males in that tank. Eggs were subsequently harvested daily and used to seed cages for rearing the GP generation. Within each replicate, equal numbers of cages were seeded from each founder tank (total number of cages: 128, 148, and 144 cages for replicates 1, 2 and 3 respectively). This ensured that each tank contributed equally to the GP generation within each replicate (see

below). The cages were distributed in randomized blocks in two environmental chambers to avoid confounding any environmental variation within chambers with possible sampling variation among founding tanks. Nymphs were reared using standard protocols (see above) and eclosing adults were removed from the cages within 24h, marked with unique numbers, separated by sex and held in stream tanks for at least seven days until formation of GP breeding units.

To form the breeding units called for in our experimental design, we adopted a stratified random sampling protocol that minimized the probability of sib matings in the P generation and ensured that the GP generation captured the variance in the source population. Our protocol ensured that each GP adult came from a unique rearing cage, the founding tanks were equally represented within each breeding unit and overall, and within each pair, the male and female came from different founding tanks.

For replicates 1 and 3, the GP generation consisted of 15 breeding units for a total of 60 mating pairs. All individuals were selected based upon their unique numbers using the following stratified random sampling scheme. We first randomly selected one number from each rearing cage, with the restriction that the final set of sampled individuals had to contain equal numbers of males and females. From this set, we formed each breeding unit by randomly selecting one male and one female originating from each of the four founding stream tanks. The resulting four males and four females were then paired such that the males and females within each pair derived from different founding stream tanks. Thus, each breeding unit consisted of eight adults from eight different rearing cages, of which one male and one female were from each of the four founding stream tanks.

For replicate 2 the GP generation consisted of six breeding units with a total of 24 mating pairs. Because we only had two founding stream tanks for GP adults, each breeding unit was set up with four males from one tank and four females from the other (all selected at random from different rearing cages as above), alternating which tank contributed which sex among the breeding units.

Each GP mating pair was maintained in a cage at room temperature. Eggs were harvested and used to seed three or four cages per female with 20 - 30 eggs each. After hatching, nymphs were redistributed among cages within dams to achieve approximate densities of 20 nymphs per cage per female. Nymphs were reared to eclosion using standard protocols as for the GP

generation, and newly eclosed adults were separated by sex and held in the laboratory for a minimum of seven days before formation of parental (P) mating sets.

Each breeding unit required four structured parental (P) mating sets consisting of three females and a single male. To maximize the probability that the male would fertilize the eggs of each of his three females, each P female occupied her own cage and the male was rotated among his three females by being transferred sequentially from one cage to another every second or third day. The egg collecting and rearing protocols for the offspring (F1) were identical to that used for the GP and P generations.

All adults were preserved in 70% ethanol prior to being photographed for measurement. F1 adults were preserved as soon as their cuticles had hardened sufficiently (typically one day post-eclosion).

G. Measurement Protocols

Our measurement protocols followed those established in previous studies of body size variation in this species (e.g., Preziosi and Fairbairn, 1996, Preziosi et al,1996, Fairbairn, 2005; Bertin and Fairbairn 2007). Each preserved individual was placed in a glass-bottomed box with a scale bar fixed to the glass. The strider was arranged with ventral side against the glass and legs spread to the sides, and held in place with a Styrofoam plug made to fit snugly into the box. The plug gently pressed the water strider against the glass. This protocol ensured that each specimen was held in a standard position with the dorso-ventral axis perpendicular to the glass and with all landmarks clearly discernable. With the specimen thus secured, the box was inverted and placed under a Leica Wild M3c dissecting microscope so that the specimen was viewed in ventral aspect for photographing. The microscope was equipped with a 0.5 reducing lens and an attached Spot Insight 3.2.0 color camera. The entire strider was photographed at 10x magnification and the posterior region was photographed again at 25x magnification.

All measurements were made from these photographs using the digitizing software SigmaScan Pro 5.0. Photographs in which landmarks were unclear were retaken. Except where noted, lengths were taken along the ventral midline. Measures of the head, thorax, abdomen and femora were taken from the photo of the entire animal (Figure S2). We measured only the right three femora unless one or more of those legs were missing or damaged (as occasionally happened during handling), in which case we substituted the three left femora. The remaining

measures were taken from the more magnified photograph of the genital region (Figure S3). The length of the margin of segment 7 was taken as the linear distance between the right lateral (outer) end of the suture between segments 6 and 7 and the tip of the connexival spine on the right side of the body. Measurements from each photograph were transformed to mm based on measurement of the included scale bar.

Unlike the somatic body components, whose lengths are fixed as adults (e.g., see Preziosi and Fairbairn, 1997), the genital segments of both sexes are mobile to facilitate copulation and oviposition (Fairbairn et al., 2003). These changes could influence our genital measures, introducing error variance and reducing heritabilities. To guard against this, all photographs of the posterior region were carefully evaluated and measurements were included only for specimens whose genitalia were fully retracted. As an additional precaution, regressions of genital measures on thorax length were used to identify outliers in which genital measures were high. In these cases, the photograph was re-evaluated and the measurement was discarded if any sign of genital extrusion was evident.

To determine our accuracy taking measurements from our photographs, we conducted a repeatability analysis for an initial set of photographs from 73 males and 167 females (n = 3 per individual. The mean intraclass correlation coefficient over all traits and both sexes was 0.966 (SD = 0.034), with a maximum of 0.996 for hind femur length in males, and a minimum of 0.878 for segment 8 length in females. The latter was the only measurement with a repeatability less than 0.9.

H. Correlations among traits

To determine if the 12 measured traits could be legitimately considered as different traits at the genetic level, we estimated the pairwise additive genetic correlations among traits within each sex using an additive-only model, with generation and replicate as fixed effects and the variance components as random effects.

All pairwise correlations in both sexes differed significantly from ±1.0, and hence none is an absolute constraint on independent evolutionary response to selection (Tables S3, S4). The magnitudes of the correlations were generally low: for male traits the mean absolute value of the 66 pairwise correlations was only 0.470 (SD 0.220), and for female traits it was only 0.324 (SD 0.258). Only five of the 132 correlations exceeded 0.8, and all of these were among the lengths

of the leg femora. Although relatively high, even these correlations were significantly less than 1.0. Many of the pairwise correlations were low (<0.3) and not significantly different from zero. This is particularly true for correlations involving SpineDist, L.Seg8 and L.Seg9.10, all traits of the terminal abdominal and genital segments. Based on these results, we included all 12 traits in our subsequent analyses.

Table S3: Between-trait additive genetic correlations within each sex (males above the diagonal, females below). Bold values differ significantly from 0. All estimates differ from ±1.0 by more than 2SE[a]. Traits are listed in the same order as in Table 1[b].

Trait	1	2	3	4	5	6	7	8	9	10	11	12
1		**0.700**	**0.660**	**0.626**	**0.524**	**0.634**	**0.481**	**0.492**	**0.528**	-0.003	**0.430**	**0.445**
2	**0.735**		**0.688**	**0.428**	**0.739**	**0.681**	**0.779**	**0.798**	**0.595**	**0.209**	0.308	**0.625**
3	**0.538**	**0.642**		**0.564**	**0.599**	**0.479**	**0.577**	**0.573**	**0.539**	**0.403**	**0.462**	**0.388**
4	**0.664**	**0.512**	**0.489**		**0.445**	**0.457**	**0.441**	**0.375**	**0.372**	0.116	**0.539**	0.302
5	**0.607**	**0.646**	**0.530**	**0.494**		**0.691**	**0.860**	**0.838**	**0.473**	**0.194**	0.253	**0.510**
6	**0.435**	**0.312**	0.051	**0.368**	**0.270**		**0.666**	**0.692**	**0.467**	-0.033	0.027	**0.653**
7	**0.658**	**0.624**	**0.537**	**0.509**	**0.893**	**0.328**		**0.966**	**0.550**	**0.189**	**0.342**	**0.605**
8	**0.654**	**0.665**	**0.531**	**0.487**	**0.793**	**0.291**	**0.954**		**0.549**	0.167	0.255	**0.666**
9	**0.402**	**0.506**	**0.639**	**0.437**	**0.385**	0.136	**0.388**	**0.379**		0.156	0.157	**0.529**
10	0.093	-0.111	0.014	**0.216**	0.036	0.113	0.069	0.016	0.090		**0.303**	-0.190
11	0.295	0.113	0.071	-0.009	0.114	-0.017	0.186	0.211	**0.349**	-0.033		0.033
12	0.238	-0.005	-0.004	-0.040	0.085	-0.057	0.078	-0.020	0.045	0.141	-0.021	

[a]See Table S4 for standard errors.
[b] Trait names: 1. Head length, 2. Thorax length, 3. Abdomen length, 4. Abdomen width, 5. Front femur length, 6. Front demur width, 7. Middle femur length, 8. Hind femur length, 9. Length of out margin of segment 7, 10. Distance between the tips of the connexival spines, 11. Length of segment 8, 12. Length of segments 9 & 10.

Table S4: Standard errors for between-trait additive genetic correlations among the 12 traits. Estimates for males are above the diagonal, females below. Traits are numbered as in Table S3.

Trait	1	2	3	4	5	6	7	8	9	10	11	12
1		0.072	0.075	0.095	0.086	0.089	0.092	0.091	0.089	0.117	0.166	0.163
2	0.101		0.054	0.091	0.045	0.068	0.041	0.038	0.064	0.094	0.156	0.115
3	0.122	0.065		0.081	0.063	0.091	0.066	0.065	0.069	0.085	0.138	0.134
4	0.121	0.087	0.088		0.088	0.102	0.089	0.095	0.095	0.107	0.151	0.162
5	0.103	0.064	0.076	0.084		0.062	0.028	0.030	0.073	0.093	0.147	0.119
6	0.163	0.133	0.140	0.126	0.121		0.069	0.065	0.093	0.115	0.190	0.126
7	0.098	0.067	0.076	0.086	0.027	0.123		0.009	0.067	0.093	0.148	0.122
8	0.096	0.061	0.073	0.084	0.039	0.123	0.013		0.066	0.092	0.152	0.110
9	0.124	0.076	0.061	0.085	0.081	0.130	0.082	0.079		0.092	0.141	0.120
10	0.147	0.108	0.107	0.101	0.101	0.136	0.103	0.099	0.096		0.137	0.146
11	0.193	0.158	0.153	0.162	0.147	0.205	0.144	0.140	0.147	0.162		0.196
12	0.167	0.135	0.130	0.138	0.124	0.170	0.123	0.121	0.120	0.120	0.154	

I. Genetic variances (as proportions of phenotypic variance) and between-sex genetic correlations for the 12 measured traits.

Table S5. Genetic variances (as proportions of the total phenotypic variance) and between-sex genetic correlations (r_A, r_a, r_d). Bold font: estimates significantly different from zero[a]. For the correlation estimates, italic font indicates significantly different from ±1. Other cases: see footnotes. Traits are numbered as in Tables S3 and S4.

Trait	h_A^2 [b]		r_A [b]	h_a^2		r_a	h_x^2		h_d^2		r_d
	M	F		M	F		M	F	M	F	
1	**0.19**	**0.07**	**0.72**	**0.19**	0.00[c]	**1.00**[d]	0.00	**0.06**	**0.31**	**0.24**	**1.00**
2	**0.49**	**0.35**	**0.96**	**0.49**	**0.31**	**1.00**	0.00	0.03	**0.30**	**0.24**	***0.66***
3	**0.58**	**0.42**	***0.81***	**0.58**	**0.31**	**0.99**	0.00	0.13	0.12	0.15	0.53
4	**0.31**	**0.33**	**0.97**	**0.31**	**0.28**	**1.00**	0.00	0.04	**0.39**	**0.29**	*0.33*
5	**0.55**	**0.43**	**0.94**	**0.55**	**0.42**	**0.97**	0.00	0.02	**0.33**	0.16	***0.71***
6	**0.22**	**0.13**	**0.97**	**0.22**	0.09	**1.00**	0.00	0.03	**0.43**	0.20	0.49
7	**0.49**	**0.43**	**1.00**	**0.47**	**0.42**	**1.00**	0.02	0.00	**0.32**	0.08	**1.00**
8	**0.52**	**0.48**	**0.97**	**0.52**	**0.44**	**1.00**	0.00	0.04	**0.32**	0.12	**1.00**
9	**0.56**	**0.52**	**0.80**	**0.56**	**0.52**	***0.80***	0.00	0.00	**0.23**	**0.21**	**1.00**
10	**0.60**	**0.50**	*0.29*	**0.60**	**0.50**	***0.29***	0.00	0.00	**0.24**	0.07	**-1.00**
11	0.05	**0.13**	**1.00**	0.05	0.00	**1.00**[d]	0.00	**0.03**	**0.95**	**0.37**	***-0.81***
12	**0.28**	**0.37**	*0.34*	**0.22**	0.19	0.54	0.06	0.09	0.29	0.04	0.08

a. Significance assessed as > 2 SE above 0 or at the boundary of 1.0 (see Table S6).
b. $h_A^2 = (V_a + V_x)/V_p$, similarly r_A contains both autosomal and sex-linked components.
c. 0.00 = estimate < 10^{-6} and set to 0.
d. We used the asreml approach that directly estimates the genetic correlation rather than attempting to separately estimating the covariances and variances. Because it uses restricted maximum likelihood the correlation cannot exceed ±1. The asreml program calculates jointly the variances and the correlations, which tends to be more stable than estimating the variances and covariances and then estimating the correlations from these. Note that the genetic correlations are based only on the genetic variances and covariances and therefore it is possible to have high genetic correlations in spite of low heritabilities (which express the genetic variances as proportions of phenotypic variances), as in these examples.
e. U: the estimated value is too small for a reliable standard error estimate.
f. NA: standard errors not estimated because correlation values were fixed at the boundary of 1.

Table S6. Standard errors for the genetic variances and correlations given in Tables S5. Traits are numbered as in Tables S3 and S4.

Trait	h_A^{2} [b]		r_A [b]	h_a^2		r_a	h_x^2		h_d^2		r_d
	M	F		M	F		M	F	M	F	
1	0.06	0.00	0.19	0.06	U[a]	NA[b]	U	0.03	0.11	0.11	NA
2	0.07	0.06	0.07	0.07	0.06	NA	U	0.03	0.11	0.11	0.26
3	0.07	0.13	0.07	0.07	0.13	0.22	U	0.10	0.10	0.10	0.50
4	0.07	0.07	0.10	0.07	0.07	NA	U	0.04	0.13	0.12	0.24
5	0.07	0.12	0.05	0.07	0.12	0.15	U	0.07	0.11	0.10	0.31
6	0.07	0.05	0.24	0.07	0.05	NA	U	0.03	0.14	0.13	0.31
7	0.06	0.06	NA	0.06	0.06	NA	0.05	0.02	0.14	0.07	NA
8	0.06	0.06	0.06	0.06	0.06	NA	U	0.02	0.11	0.07	NA
9	0.06	0.06	NA	0.06	0.06	0.06	U	U	0.10	0.09	NA
10	0.07	0.06	0.06	0.07	0.06	0.10	U	U	0.11	0.07	NA
11	0.06	0.00	0.10	0.06	U	NA	U	0.01	0.06	0.17	0.21
12	0.09	0.18	0.19	0.09	0.18	0.39	0.10	0.07	0.20	0.13	1.05

a. U: the estimated value is too small for a reliable standard error estimate.
b. NA: standard errors not estimated because correlation values were fixed at the boundary of 1.

J. References

Bertin A, Fairbairn DJ (2005). One tool, many uses: precopulatory sexual selection on genital morphology in *Aquarius remigis*. J Evol Biol 18(4)**:** 949-961

Blanckenhorn WU, Preziosi RF, Fairbairn DJ (1995). Time and energy constraints and the evolution of sexual size dimorphism - to eat or to mate? Evol Ecol 9:369-381

Calabrese DM (1974) Population and subspecific variation in *Gerris remigis* Say. Entomol News 85:27-28

Carbonell EA, Frey JJ, Bell AE* (1985) Estimation of maternal, sex-linked and additive x additive epistatic gene effects for body size of *Tribolium*. Theor Appl Genet 70:133-137

Cotter SC, Kruuk LE, Wilson K* (2004) Costs of resistance: genetic correlations and potential trade-offs in an insect immune system. J Evol Biol 17:421-429

Cowley DE, Atchley WR (1988) Quantitative genetics of *Drosophila-melanogaster*. 2. Heritabilities and genetic correlations between sexes for head and thorax traits. Genetics 119:421-433

Cowley DE, Atchley WR, Rutledge JJ (1986) Quantitative genetics of *Drosophila melanogaster*. I. Sexual dimorphism in genetic parameters for wing traits. Genetics 114:549-566

Evans SR, Schielzeth H, Forstmeier W, Sheldon BC, Husby A* (2014) Nonautosomal genetic variation in carotenoid coloration. Am Nat 184:374-383

Fairbairn DJ (1986) Does alary polymorphism imply dispersal dimorphism in the waterstrider, *Gerris remigis*? Ecol Ent11:355-368

Fairbairn DJ (1992) The origins of allometry: size and shape polymorphism in the common waterstrider, *Gerris remigis* Say (Heteroptera, Gerridae). Biol J Linn Soc 45:167-186

Fairbairn DJ (2005) Allometry for sexual size dimorphism: Testing two hypotheses for Rensch's rule in the water strider, *Aquarius remigis.* Am Nat 166: S69-S84

Fairbairn DJ, Desranleau L (1987) Flight threshold, wing muscle histolysis, and alary polymorphism: correlated traits for dispersal tendency in the Gerridae. Ecol Ent 12:13-24

Fairbairn DJ, King E (2009) Why do Californian striders fly? J Evol Biol 22:36-49

Fairbairn DJ, Vermette R, Kapoor NN, Zahiri N (2003) Functional significance of sexually selected genitalia in the water strider, *Aquarius remigis*. Can J Zool 81: 400-413

Fox CW (1994)* Maternal and genetic influences on egg size and larval performance in a seed beetle (*Callosobruchus maculatus*): multigenerational transmission of a maternal effect? Heredity 73:509-517

Fox CW, Savalli UM (1998) Inheritance of environmental variation in body size: Superparasitism of seeds affects progeny and grandprogeny body size via a nongenetic maternal effect. Evolution 52:172-182

Griffin, RM, Schielzeth H, Friberg U (2016) Autosomal and x-linked additive genetic variation for lifespan and aging: Comparisons within and between the sexes in *Drosophila melanogaster*. G3-Genes Genomes Genet. 6:3903-3911

Husby A, Schielzeth H, Forstmeier W, Gustafsson L, Qvarnstrom A (2013). Sex chromosome linked genetic variance and the evolution of sexual dimorphism of quantitative traits. Evolution 67(3)**:** 609-619

Kaitala A, Dingle H (1992) Spatial and temporal variation in wing dimorphism of California populations of the waterstrider *Aquarius remigis*. Ann Entomol Soc Am 85:590-595

Kaufmann P, Wolak ME, Husby A, Immonen E (2021). Rapid evolution of sexual size dimorphism facilitated by Y-linked genetic variance. Nat Ecol Evol 5:1394-1402

Keena MA, Grinberg PS, Wallner WE (2007)* Inheritance of female flight in *Lymantria dispar* (Lepidoptera: Lymantriidae). Environ Entomol 36:484-494

Kosova G, Abney M, Ober C (2010) Colloquium papers: Heritability of reproductive fitness traits in a human population. Proc Nat Acad Sci USA 107 Suppl 1:1772-1778

Larsen CT, Holand AM, Jensen H, Steinsland I, Roulin A (2014) On estimation and identifiability issues of sex- linked inheritance with a case study of pigmentation in Swiss barn owl (*Tyto alba*). Ecol Evol 4:1555-1566

Mezey JG, Houle D (2005)* The dimensionality of genetic variation for wing shape in *Drosophila melanogaster*. Evolution 59:1027-1038

Mousseau TA, Dingle H (1991) Maternal effects in insect life histories. Ann. Rev. Ent. 36:511-534

Pan L, Ober C, Abney M (2007) Heritability estimation of sex-specific effects on human quantitative traits. Genet Epidemiol 31:338-347

Polhemus JT, Chapman HC (1979) Family Gerridae: water striders, pond skaters, wherrymen. Pp. 58-69 *in* Menke, A. S., ed. The Semiaquatic and Aquatic Hemiptera of California. Bull Cal Insect Survey Vol 9. University of California Press, Berkeley

Preziosi RF, Fairbairn DJ (1996) Sexual size dimorphism and selection in the wild in the waterstrider *Aquarius remigis*: Body size, components of body size and male mating success. J Evol Biol 9: 317-336

Preziosi RF, Fairbairn DJ (1997) Sexual size dimorphism and selection in the wild in the waterstrider *Aquarius remigis*: Lifetime fecundity selection on female total length and its components. Evolution 51(2)**:** 467-474

Preziosi RF, Fairbairn DJ, Roff DA, Brennan JM (1996) Body size and fecundity in the waterstrider *Aquarius remigis*: A test of Darwin's fecundity advantage hypothesis. Oecologia 108: 424-431

Roff DA, Sokolovska N[*] (2004) Extra-nuclear effects on growth and development in the sand cricket *Gryllus firmus*. J Evol Biol 17:663-671

Roulin A, Altwegg R, Jensen H, Steinsland I, Schaub M (2010)[*] Sex-dependent selection on an autosomal melanic female ornament promotes the evolution of sex ratio bias. Ecol Lett 13:616-626

Roulin A, Jensen H (2015)[*] Sex-linked inheritance, genetic correlations and sexual dimorphism in three melanin-based colour traits in the barn owl. J Evol Biol 28:655-666

Tringali A, Bowman R, Husby A (2015)* Selection and inheritance of sexually dimorphic juvenile plumage coloration. Ecol Evol 5:5413-5422

Willmore KE, Roseman CC, Rogers J, Richtsmeier JT, Cheverud JM (2009) Genetic variation in baboon craniofacial sexual dimorphism. Evolution 63:799–806

Wittenburg D, Teuscher F, Reinsch N[*] (2011) Statistical tools to detect genetic variation for a sex dimorphism in piglet birth weight. J Anim Sci 89:622-629

Wolak ME, Keller LF (2014) Dominance genetic variance and inbreeding in natural populations. In: Charmantier A, Garant D, Kruuk LEB (eds) Quantitative Genetics in the Wild. Oxford Univ Press, New York, pp104-127

Zhu J, Weir BS[*] (1996) Diallel analysis for sex-linked and maternal effects. Theor Appl Genet 92:1-9
